# Baseline Assessment of Poultry Production, Pharmaceutical Product Use, and Related Challenges on Commercial Poultry Flocks in Kano and Oyo States of Nigeria

**DOI:** 10.3390/vetsci8120315

**Published:** 2021-12-08

**Authors:** Isabella C. Endacott, Erika Galipo, Abel B. Ekiri, Ruth Alafiatayo, Kehinde Adebowale, Mariana Dineva, Aliyu Wakawa, Adah Ogwuche, Beatty-Viv Maikai, Bryony Armson, Erik Mijten, Gabriel Varga, Alasdair J. C. Cook

**Affiliations:** 1 Department of Veterinary Epidemiology and Public Health, School of Veterinary Medicine, University of Surrey, Guildford GU2 7AL, UK; I.Endacott@liverpool.ac.uk (I.C.E.); erika.galipo@apha.gov.uk (E.G.); r.alafiatayo@surrey.ac.uk (R.A.); mariana.dineva@surrey.ac.uk (M.D.); b.armson@surrey.ac.uk (B.A.); 2 School of Veterinary Science, University of Liverpool, Neston CH64 7TE, UK; 3 Zoetis Belgium S.A., 1930 Zaventem, Belgium; kehinde.adebowale@zoetis.com (K.A.); adah.ogwuche@zoetis.com (A.O.); erik.mijten@zoetis.com (E.M.); gabriel.varga@zoetis.com (G.V.); 4 Department of Veterinary Medicine, Ahmadu Bello University, Zaria 810211, Nigeria; asmwakawa@yahoo.com; 5 Department of Veterinary Public Health and Preventative Medicine, Ahmadu Bello University, Zaria 810211, Nigeria; beatt18@gmail.com (B.-V.M.); alasdair.j.cook@surrey.ac.uk (A.J.C.C.)

**Keywords:** poultry, poultry health, management practices, challenges, poultry diseases, Nigeria, Africa

## Abstract

Poultry production is a major component of the livestock sector in Nigeria and continues to expand rapidly; however, it is still constrained by low productivity. A farm survey was conducted to provide a baseline assessment of poultry production (products generated, farm costs, and revenue), pharmaceutical use, and related challenges faced by farmers on 44 commercial poultry farms in Oyo and Kano states of Nigeria. Live spent layers, eggs, and used beddings were the most frequently sold products for revenue. Antibiotic products were widely used, the most reported were Doxygen, Tylosin, and Conflox. Overall, 40% of farms used feed additives (including toxin binders, minerals, and vitamins) and 12% used coccidiostats. Access to pharmaceutical products was a key challenge and appeared to disproportionally affect farmers in the northern part (Kano) of Nigeria. Other challenges included perceived antibiotic ineffectiveness, high cost of drugs, and long distances to pharmaceutical suppliers. Challenges related to vaccine use were unavailability, distance to the supplier, and health issues interfering with the vaccination schedule. Study findings highlight the need for improved access to veterinary pharmaceuticals, particularly in the northern states. Further investigations into the causes of antibiotic ineffectiveness and strategies for distribution of high-quality, effective pharmaceuticals are also necessary.

## 1. Introduction

Despite its declining contribution to Nigeria’s foreign exchange earnings, agriculture continues to be the most important sector of the Nigerian economy; it provides employment and an estimated 65% of Nigerians depend on agriculture for their livelihood [1]. Nigeria’s poultry population of nearly 200 million birds [2] makes poultry production the most commercialised agricultural livestock sector in the country [3]. The majority of the medium- and large-scale poultry farms are located in the urban and peri-urban areas of southwest Nigeria, but the north has a larger share of small farmer holdings located in rural or peri-urban areas compared to the south [4]. 

Poultry diseases negatively affect the poultry industry and alongside effective prevention measures, the availability and access to appropriate preventive and treatment options is critical to reducing productivity and economic losses. However, there are limited data available on pharmaceutical products used for disease prevention and treatment on commercial poultry farms in Nigeria. A few previous studies focussed only on antibiotic use on poultry farms in Ogun [5,6] and Kwara states [7]. Data on the overall pharmaceutical products and the challenges related to pharmaceutical product availability and use experience of poultry farmers are lacking. In addition, despite the size of the poultry sector in Nigeria, data on poultry products produced on the farm, farm costs, and revenue are also still limited. Together, these data would be useful to the poultry industry and policy makers in identifying appropriate interventions and in guiding decisions and policy aimed at improving poultry productivity. 

The aim of the current study was to improve our understanding of poultry production (including products generated on farm for revenue and related farm costs), pharmaceutical product use, and the related challenges on commercial poultry farms in Kano and Oyo states of Nigeria. 

## 2. Materials and Methods

### 2.1. Ethics

The institutional ethical review and approvals were granted by the Ahmadu Bello University, Nigeria, Ethical committee (Ethics approval number: ABUCAUC/2018/055) and by the University of Surrey, United Kingdom, Animal Welfare and Ethical Review Board (NASPA Reference: NERA-1819-003).

### 2.2. Study Population and Area

This study targeted poultry farms that reared birds for commercial purposes, including broilers, layers, and breeders. The farms included in this study were located in Oyo (in the southwest geopolitical zone) and Kano (in the northcentral geopolitical zone) states in Nigeria. These states were selected using purposive sampling approach; a map showing the geographical location of the selected states and information on the state selection criteria have been described in detail elsewhere [8].

### 2.3. Study Approach

This study was part of a larger project in commercial poultry farms in Kano and Oyo states of Nigeria, which includes two further related papers that: (i) examined baseline parameters on poultry health, management practices, and the related challenges [9], and (ii) investigated flock protection based on antibody titres and characterised circulating strains of Newcastle disease virus (NDV), infectious bursal disease virus (IBDV), and infectious bronchitis virus (IBV) strains in the poultry flocks [8].

The current study was a cross-sectional survey amongst commercial poultry farms that collected baseline data (between 13 May 2019, and 8 June 2019) on poultry production (including products generated on farm for revenue and related farm costs), pharmaceutical product use and the related challenges experienced by these farmers. In total, 44 farms were randomly selected (16 in Oyo and 28 in Kano) using probability-proportional-to-size sampling, where the number of farms selected in each state were proportionate to the number of farms present in the state. Detailed information about the sampling approach and the sample size calculation for the farms has been provided elsewhere [8].

### 2.4. Data Collection

A questionnaire (Questionnaire S1) was used to collect relevant data on tablet computers using Qualtrics software (Version: March 2020; Provo, UT, USA). Data on the following parameters were collected: farm characteristics, production, management/husbandry practices, general use of pharmaceutical products on-farm, farm-level financial costs and revenue, and the challenges faced. All questions are presented in tables, figures, or Supplementary Data.

### 2.5. Data Analysis

Survey results were downloaded to Microsoft Excel (MSO: 16.0.14131.20358) and data were cleaned. Descriptive statistics were performed to summarise the data for the parameters related to poultry production, farm costs and revenue, pharmaceutical product use, and the associated challenges.

## 3. Results

### 3.1. Farm Characteristics and Respondent Demographics

A total of 44 farms participated in this study, including 28 in Kano state and 16 in Oyo state. One farm in Kano had an incomplete dataset because data could not be retrieved from the tablet, and it was therefore excluded from the data analysis. 

Respondent demographics and farm information are presented in Table S1. Briefly, the study respondents were mostly farm owners (29/43; 67.4%) or farm managers (16/43; 37.2%), male (30/42; 71.4%), and most were aged between 46–60 years (20/42; 47.6%) or between 35–45 years (12/42; 28.6%). Over a third (16/43; 37.2%) of respondents had 11–20 years’ experience in poultry farming and 27.9% (12/43) had between 6–10 years of experience. At the time of the study visit and survey administration, most participating farms kept layers only (21/43; 48.8%), followed by layers and broilers (12/43; 27.9%), and broilers only (9/43; 20.9%).

### 3.2. Poultry Production

#### 3.2.1. Products Obtained from the Farm and Sold to Generate Revenue

**Figure 1:**
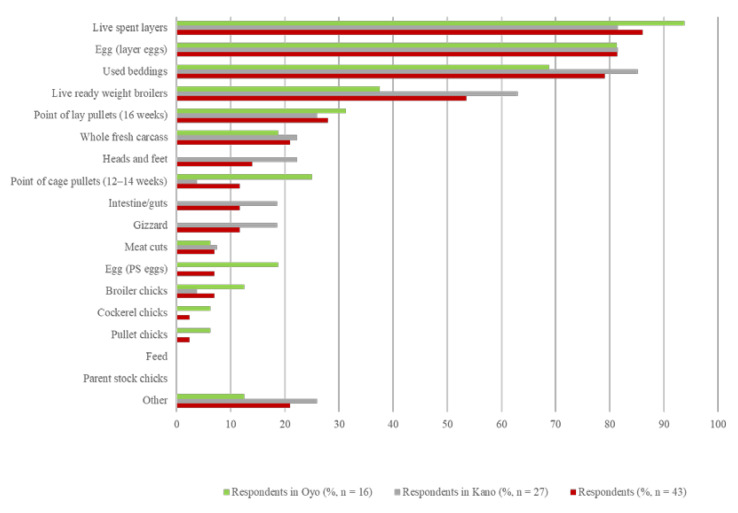
Products obtained from poultry farms and sold by farmers to generate revenue (*n* = 43). Respondents selected all applicable products sold; percentages in total and by state do not add up to 100%. Other (*n* = 9) includes manure and sacks (*n* = 1), imported cages (*n* = 1), used feed bags (*n* = 6), and point of lay pullets (at 14 to 16 weeks) (*n* = 1). PS, parent stock.

The three most frequently reported poultry products obtained and sold to generate income by respondents in Oyo and Kano were live spent layers (sold as live birds at the end of production cycle) (37/43; 86.1%), followed by eggs (35/43; 81.4%), and used beddings (34/43; 79.1%). In Kano, the product most frequently sold to generate income was used beddings (23/43; 85.2%), whereas the top product in Oyo was live spent layers (15/17; 93.7%). All types of poultry farm products sold to generate revenue in total and by state are illustrated in Figure 1. 

On layer only farms, the most reported revenue generating poultry products were live spent layers (21/21; 100%), eggs (20/21; 95.2%) and used beddings (17/21; 91.0%). On broiler only farms, live ready-weight broilers (8/9; 88.9%), used beddings (6/9; 66.7%) and whole fresh carcasses (4/9; 44.4%) were the most sold products for revenue. On mixed (layer and broiler) farms, live spent layers (12/12; 100%), eggs (12/12; 100%), and used beddings (11/12; 91.7%) were mostly reported as products sold for revenue.

#### 3.2.2. Products Perceived to Generate the Highest Revenue

**Figure 2:**
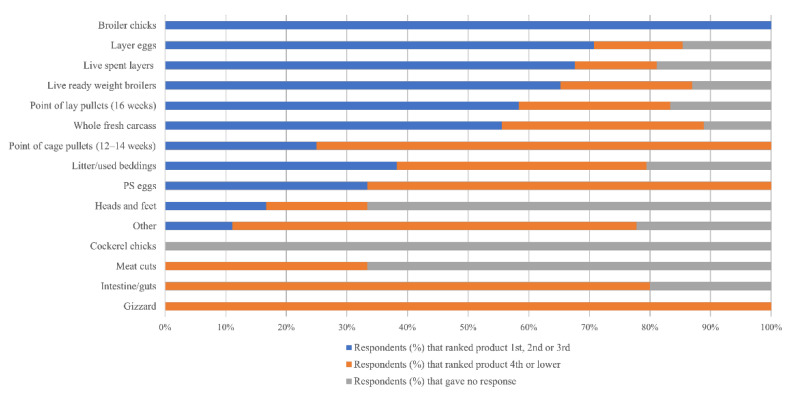
Products sold by farmers ranked on perceived revenue generated. First rank denotes the top revenue generating product (i.e., rank 1, 2 or 3). Low rank (i.e., rank 4, 5, 6) denotes a lower revenue generating product. PS, parent stock.

Respondents were asked to rank all poultry farm products sold to generate revenue (as specified above) from most to least important with regards to generating income. The products that were most consistently ranked within the top three were broiler chicks (3/3; 100.0%), eggs (29/35; 82.9%), live spent layers (25/37; 67.6%) and live ready-weight broilers (15/23; 65.2%) (Figure 2). 

On layer only farms, eggs (17/20; 85.0%), live spent layers (16/21; 76.2%) and point-of-lay pullets (5/8; 62.5%) were the poultry products most ranked in the top three money-generating products. On broiler only farms, the products sold that were most often ranked in the top three were broiler chicks (1/1; 100%), eggs (3/3; 100%), point-of-lay pullets (1/1; 100%) and used beddings (5/6; 83.3%). On mixed farms, parent stock (PS) eggs (1/1; 100%), broiler chicks (1/1; 100%), and eggs (9/12; 75.0%) were the poultry products most often ranked in the top three for generating money.

#### 3.2.3. Secondary Activities Performed on the Farm to Generate Revenue

Twelve respondents (12/43; 27.9%) reported processing farm waste into manure for crops, while 10/43 (23.3%) operated a private feed mill to produce feed for on-farm use. Additionally, one respondent (2.3%) operated a commercial feed mill, and two respondents (4.7%) had a slaughterhouse onsite. 

### 3.3. Farm Costs and Revenue in the Previous Production Cycle

#### 3.3.1. Number of Chicks on Farm

Overall, the reported median number of broiler chicks at the beginning of the previous production cycle was 500 (min = 100, max = 5000, *n* = 15). Respondents in Oyo reported a higher median number of broiler chicks (median = 2350, min = 200, max = 5000, *n* = 4) than those in Kano (median = 500, min = 100, max = 2500, *n* = 11).

Overall, the reported median number of layer chicks at the beginning of the previous production cycle was 4000 (min = 300, max = 26,000, *n* = 35). As with the results on broiler chicks, Oyo respondents reported a higher median number of layer chicks (median = 4377, min = 1500, max = 12,960, *n* = 13) than Kano (median = 3250, min = 300, max = 26,000, *n* = 22). 

In total, the reported median number of PS chicks at the beginning of the previous production cycle was 5500 (min = 4000, max = 7000, *n* = 2); these were only reported in Oyo. Cockerel chicks were also kept in both Oyo and Kano farms (median = 100, min = 20, max = 500, *n* = 5), but a higher median number was reported in Kano (median = 150, min = 50, max = 500, *n* = 4) compared to Oyo (median = 20, min = 20, max = 20, *n* = 1) (Table S2).

#### 3.3.2. Cost of One-Day Old Chicks

Across both states, the reported median cost of one-day old broiler, layer, and cockerel chicks purchased for raising on farm in the previous production cycle was 270 Nigerian Naira (NGN) (min = 120, max = 920, *n* = 15), 200 NGN (min = 120, max = 65,000, *n* = 31), and 30 NGN (min = 25, max = 1000, *n* = 5), respectively ( 1 US dollar = 411.400 NGN at the time of writing in August 2021) (Table S3). 

#### 3.3.3. Number of Chicks Hatched at Hatchery on Farm

Only two farms reported having hatcheries on site and both were located in Oyo. Both farms hatched 10,000 broiler chicks each, while on one of the two farms, the number of pullet (layer) chicks hatched was 15,000 and the number of cockerel chicks hatched was 10,000 (Table S4). 

#### 3.3.4. Source of Day-Old Chicks (DOCs) for Farms with No Hatchery

Of the 41 farms that did not own a hatchery on site or in another location, almost all (39/41; 90.8%) sourced their DOCs (broiler and layer chicks) from commercial producers. Two (4.7%) respondents did not specify the source of chicks.

#### 3.3.5. Production Information on Broiler Birds Reared on Farm

The reported median number of broiler birds that reached slaughter weight in the previous production cycle on study farms in Oyo and Kano was 200 (min = 35, max = 2940, *n* = 13); this number was higher in Oyo (median = 1929, min = 43, max = 2940, *n* = 3) than in Kano (median = 200, min = 35, max = 450, *n* = 10) (Table 1).

Overall, the reported median age of broiler birds at slaughter weight in the previous production cycle was 6.5 weeks (min = 5, max = 10, *n* = 16) (Table 1). In total, the reported median slaughter weight in the previous production cycle was 1.8 kg (min = 1.2, max = 6.0, *n* = 12); the slaughter weight was slightly higher in Oyo (median = 2 kg, min = 1.8, max = 2.5, *n* = 4) than in Kano (median = 1.4 kg, min = 1.2, max = 6, *n* = 8).

Overall, the reported median price for a broiler bird at the weight ready for slaughter in the previous production cycle was 1300 NGN (min = 530, max = 3500, *n* = 16); the price was higher in Oyo (median = 1850, min = 530, max = 3500, *n* = 6) than in Kano (median = 1300, min = 800, max = 1700, *n* = 10).

In total, the median number of reported dead broiler birds on the farms in the previous production cycle was 50 birds (min = 7 birds, max = 2800, *n* = 16); mortality was higher in Oyo by number of birds (median = 85, min = 7, max = 180, *n* = 5) compared to Kano (median = 30, min = 10, max = 2800, *n* = 11) (Table 1). The number of unproductive broiler birds culled from the farms in Oyo and Kano are shown in Table S5. 

#### 3.3.6. Production Information on Layer Birds Reared on Farm

The reported median number of layer birds that reached maturity in the previous production cycle in Oyo and Kano was 2797 (min = 90, max = 56,000, *n* = 30) (Table 2); this was higher in Oyo, (median = 3210, min = 160, max = 11,800, *n* = 12) than in Kano (median = 993, min = 90, max = 56,000, *n* = 18).

Overall, the median reported length of the production cycle of layer birds was 80 weeks (min = 20, max = 130, *n* = 31), higher in the Oyo birds (median = 88, min = 63, max = 114, *n* = 13) than in the Kano birds (median = 72, min = 20, max = 130, *n* = 18). The median average price per crate of eggs sold was 800 NGN (min = 400, max = 900, *n* = 32) and it was similar in both states (Table 2). 

The median number of spent-layers or off-layer birds sold for slaughter in Oyo and Kano was 2440 birds (min = 80, max = 51,000, *n* = 32); 3750 birds (min = 800, max = 14,250, *n* = 13) in Oyo, and 2100 birds (min = 80, max = 51,000, *n* = 19) in Kano (*p* = 0.081). Overall, the median price of spent-layers or off-layer birds sold for slaughter was 1200 NGN (min = 800, max = 1500, *n* = 33); the reported price was higher in Oyo (median = 1250, min = 1100, max = 1500, *n* = 13) than in Kano (median = 1150, min = 800, max = 1350, *n* = 20).

The reported median number of dead layer birds in the previous production cycle in Oyo and Kano was 290 birds (min = 7, max = 5000, *n* = 32). The reported mortality was slightly lower in Oyo (median = 278, min = 7, max = 3900, *n* = 13) than in Kano (median = 340, min = 10, max = 5000, *n* = 19). The number of unproductive layer birds culled from the farm are shown in Table S5. 

#### 3.3.7. Estimated Farm Management Costs

Overall, respondents in Oyo and Kano reported the following estimated median costs related to farm management during the previous production cycle: 260,000 NGN (min = 32,000, max = 1650,000, *n* = 11) for feeding broiler birds, 4270,500 NGN (min = 1379,000, max = 49,275,000, *n* = 11) for feeding layer birds, 75,000 NGN (min = 30, max = 1680,000, *n* = 15) on antibiotic treatments, 56,000 NGN on vaccination programmes (min = 3200, max = 750,000, *n* = 21), 13,500 NGN (min = 2000, max = 364,000, *n* = 32) to maintain biosecurity on farm, 20,000 NGN (min = 2000, max = 10,800,000, *n* = 27) on litter management, and 540,000 NGN (min = 20,000, max = 18,000,000, *n* = 39) on staff wages and benefits (Table 3). 

#### 3.3.8. Estimated Vaccines Cost

All farms in this study reported vaccinating their birds in the previous production cycle against Newcastle disease (ND), infectious bursal disease (IBD), and fowl pox. The estimated median costs reported for vaccinating 1000 birds with the most administered vaccines ND, IBD and fowl pox during the previous production cycle were 2500 (min = 950, max = 26,200, *n* = 35), 1750 (min = 600, max = 5000, *n* = 32) and 2200 (min = 500, max = 8000, *n* = 27), respectively. The cost of ND vaccines per 1000 birds reported by Kano farmers was higher than that reported by Oyo farmers (median: 4900 vs 1000 NGN). Full details on vaccination costs are provided in Table S6, and more details regarding other vaccinations administered on these farms can be found in the related paper that examined baseline parameters on poultry health, management practices, and the related challenges [9].

### 3.4. Pharmaceutical Products Used on the Farm in Previous Production Cycle

#### 3.4.1. Antibiotics Administered

**Figure 3:**
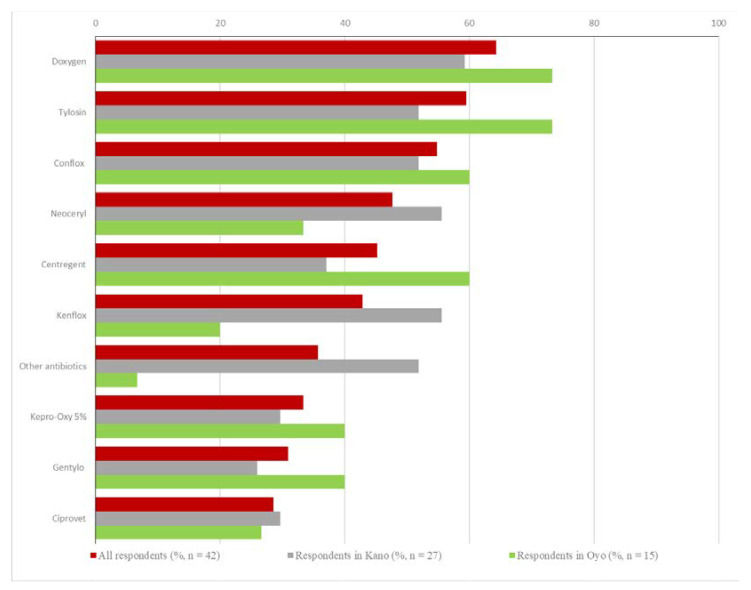
The 10 most frequently administered antibiotic products on poultry farms during the previous cycle as reported by the participants. Respondents selected all applicable types of antibiotic product; percentages in total and by state do not add up to 100%. Doxygen: Doxycycline & Gentamicin, Conflox: Enrofloxacin, Neoceryl: Neomycin and Multivitamins, Centregent: Gentamicin, Kenflox: Enrofloxacine, Kepro-Oxy 5%: Oxytetracycline, GenTylo: Gentamycin and Tylosin, Ciprovet: Ciprofloxacin.

All respondents but one (42/43, 97.7%) reported administering antibiotics during the previous production cycle. The three most frequently reported antibiotics were Enrofloxacin (Conflox: 23/42, 54.8%; Kenflox: 18/42, 42.9; Bioflox: 11/42, 26.2; Floxinol: 7/42, 16.7: Enrovet: 3/42, 7.1%: Enrocoli-Max: 2/42, 4.8%), Gentamicin (Doxygen: 27/42, 64.3: Centregent: 19/42, 45.2%; GenTylo: 13/42, 31.0%), and Tylosin (Tylosin: 25/42, 59.5%; GenTylo: 13/42, 31.0%) (Figure 3). On layer farms, the most used products were Tylosin (tylosin) (12/20; 60.0%), and Doxygen (doxycycline and gentamicin) (11/20; 55.0%), followed by Conflox (enrofloxacin), Neoceryl (neomycin and multivitamins), and Kenflox (enrofloxacine) (each used by 10/20; 50.0%). On broiler farms, the most used antibiotic product was Doxygen (doxycycline and gentamicin) (7/9; 77.8%), followed by Tylosin (tylosin), Conflox (enrofloxacin), and Gentylo (gentamycin and tylosin) (each used by 6/9; 66.7%). On mixed farms, the antibiotic product most widely used was also Doxygen (doxycycline and gentamicin) (8/12; 66.7%), followed by Tylosin (tylosin) and Conflox (enrofloxacin) (each used by 6/12; 50.0%). The complete list of antibiotic products used in total and by state is provided in Table S7. The most common duration of antibiotic product use reported was 3–5 days for all three of the most frequently used antibiotic products overall that were mentioned above (Table S8).

#### 3.4.2. Provision of Supplementary Additives in Bird Feed

**Figure 4:**
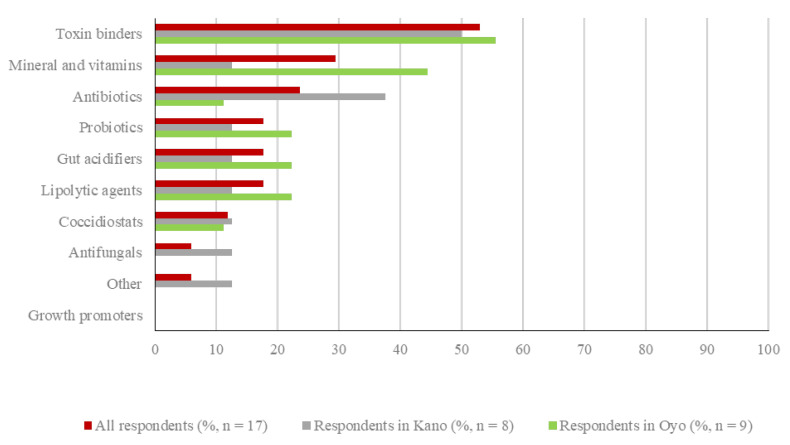
Type of medication added to poultry feed in the previous production cycle in total and by state. Respondents selected all applicable types of medication they used.

Seventeen (17/43; 39.5%) respondents in Oyo and Kano reported providing feed mixed with medications or other additives to their birds in the previous cycle. Over half of Oyo respondents (9/16; 56.3%) provided supplemented feed compared to less than a third of Kano respondents (8/27; 29.6%) (Figure 4). 

Antibiotic products were added to feed by four of these respondents (23.5%), of whom, one respondent added Zinc Bacitracin and Oxynil RX Dry, one respondent added neotreat, and two respondents could not remember the type of antibiotic they had added. Regarding the type of feed to which antibiotics were added, two respondents mixed the antibiotics with layer feed, while one respondent added antibiotics to both broiler starter and layer grower feed. 

Apart from antibiotics, other feed additives most used by the 17 respondents in Oyo and Kano were toxin binders (9/17; 52.9%), followed by minerals and vitamins (5/17; 29.5%). Coccidiostats were added to feed by two respondents only (2/17; 11.8%), one in each state. Full details on the type of poultry feed additives used in total and by state are provided in Figure 4.

#### 3.4.3. Source of Pharmaceutical Products Used

Overall, pharmaceutical products (including drugs, vaccines, and diagnostic tools) used on farms were mainly sourced from veterinary shops (36/43, 83.7%); this was the case in Kano (25/27, 92.6%) and in Oyo (11/16, 68.7%). The next most popular source was veterinary pharmaceutical distributors (8/43, 18.6%) in both Oyo (4/16, 25.0%) and in Kano (4/27, 14.8%). Veterinarians were used the least—by 4.7% only (Table 4). 

All these sources (i.e., the veterinary shops, pharmaceutical distributers, and veterinarians) were located in towns or cities; veterinary shops (35/35, 100.0%), pharmaceutical distributors (8/8, 100.0%) and veterinarians (2/2, 100.0%). The most reported estimated travel time to the source location was less than an hour (21/43, 48.8%) and between one and two hours (18/43, 41.9%) (Table 5).

#### 3.4.4. Source of Vaccines, Advice on Vaccination Protocols and Person Performing Vaccinations

Respondents most commonly sourced their vaccines from vet shops (32/43, 74.4%), and reported receiving advice on vaccination protocols mainly from their veterinarian (16/43, 37.2%). In Kano, respondents received advice on vaccination protocols primarily from government officials (12/27, 44.4%), followed by veterinarians (8/27, 29.6%), while in Oyo, veterinarians (8/16, 50%) were the most common source of advice, followed by 6/16 (37.5%) farmers that used their own knowledge. Most of the vaccinations on the farm were performed by the farm owner or a farm worker (29/43, 67.4%), or the contracted vet (17/43, 39.5%). Full details on these vaccination parameters are provided in Table S9.

#### 3.4.5. Challenges Experienced in Relation to Pharmaceutical Products

**Figure 5:**
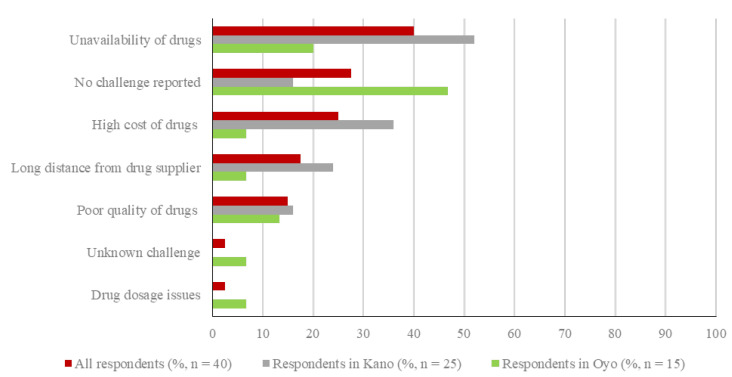
Overall challenges related to the availability of pharmaceutical products on-farm, as indicated by the respondents. Respondents selected all applicable challenges; percentages in total and by state do not add up to 100%.

Overall, the three most frequently perceived challenges related to the availability of pharmaceutical products needed on the farms were the unavailability of drugs (16/40, 40.0%), the high cost of drugs (10/40, 25.0%) and the long distance from the location of the drug supplier (7/40, 17.5%) (Figure 5). In Oyo, the most frequently reported challenges were the unavailability of drugs (3/15, 20%) and the poor quality of drugs (as perceived by farmers based on response after administration, 2/15, 13.3%). In Kano, the unavailability of drugs (13/25, 52%), the high cost of drugs (9/25, 36%), and the long distance from the location of drug supplier (6/25, 24%) were the main challenges faced by respondents (Figure 5). 

**Figure 6:**
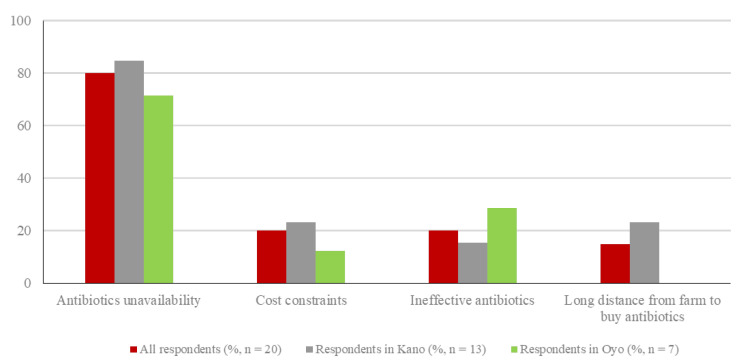
Challenges experienced when obtaining pharmaceutical products needed for farm use, as indicated by the respondents. Respondents selected all applicable challenges; percentages in total and by state do not add up to 100%.

Nearly half of the respondents (20/43; 46.5%) experienced challenges when obtaining pharmaceutical products (including drugs, vaccines, and diagnostic devices) needed for farm use and 53.5% (23/43) of the respondents did not face challenges. The reported perceived challenges included antibiotic unavailability (16/20, 80.0%), cost constraints (4/20, 20.0%), ineffective antibiotics (4/20, 20.0%), and long distance to antibiotic source from the farm (3/20, 15.0%) (Figure 6). The proportion of respondents that reported experiencing challenges in obtaining pharmaceutical products was slightly higher in Kano (13/27, 48.1%) than in Oyo (7/16, 43.8%). Antibiotic unavailability was the most frequently reported challenge in both Kano (11/13, 84.6%) and Oyo (5/7, 71.4%) (Figure 6).

**Figure 7:**
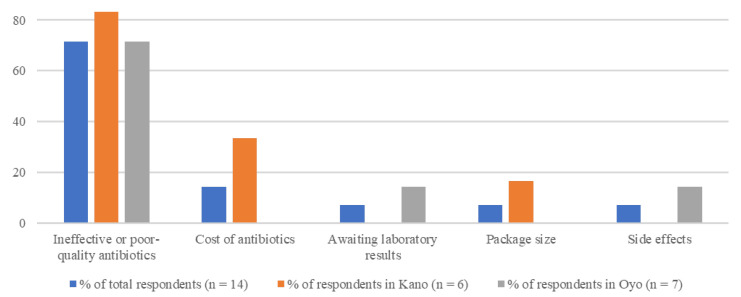
Challenges related to the use of pharmaceutical products on poultry farms, as indicated by the respondents. Respondents selected all applicable challenges; percentages in total and by state do not add up to 100%.

Over a third of the respondents (15/43; 34.9%) faced challenges while using pharmaceutical products on the farm and 62.8% (27/43) did not experience challenges. One respondent in Oyo reported experiencing these challenges but did not specify the challenge; total respondents for the analysis by challenge type was therefore 14. The main reported challenges perceived by the respondents were ineffective or poor-quality antibiotics (10/14, 71.4%) and cost of drugs (2/14, 14.3%) (Figure 7). Within those who answered the question on challenges when using pharmaceuticals on the farm, the proportion of respondents that reported experiencing these challenges was higher in Oyo (8/16, 50.0%) than in Kano (7/26, 26.9%). Ineffective or poor-quality antibiotics were the most frequently reported challenge in both Oyo (5/7, 71.4%) and Kano (5/7, 71.4%) (Figure 7).

#### 3.4.6. Challenges Experienced in Relation to Vaccines

Overall, the most described challenges related to the vaccines included health issues interfering with the schedule (7/42; 16.7%), unavailability of vaccines (6/42; 14.3%), and long distance from location of vaccine supplier (6/42; 14.3%) (Table 6).

## 4. Discussion

The findings from this study provide useful information on poultry production, farm costs and revenue, pharmaceutical product use, and the associated challenges faced by farmers on commercial broiler and layer farms in Oyo and Kano states of Nigeria. These main themes investigated in this study are discussed, as well as the study limitations and the recommendations based on the findings.

### 4.1. Poultry Production

#### 4.1.1. Hatcheries

The current study revealed that it was uncommon for poultry farmers to have their own hatcheries (i.e., located on the farm). Other studies have found a similar trend, with most poultry farmers purchasing their rearing stock from live bird markets rather than directly from hatcheries [10]. In this study, 91% of the respondents who did not have their own hatcheries sourced their DOCs from external commercial hatcheries with minimal on-farm DOC hatching. Farmers within an area have been reported to often source their chicks from the same commercial distributer, suggesting the existence of large outlets that provide DOCs for specific regions [11]. However, farmers have reported lack of standard controls or rules in the existing hatcheries, which allow for selling of low-quality and unhealthy DOCs [12]. To ensure DOC outlets and sellers meet quality standards, the establishment of nationwide standard guidelines stemming from the beginning of the poultry-production chain are necessary, such as those by the Red Tractor Assurance in the United Kingdom [13]. 

#### 4.1.2. Poultry Products

The two main products sold by farmers to generate income were live spent layers, and eggs; this finding reflects that predominantly layer birds were reared on these farms. In the current study, the higher number of layer producers compared to those rearing broilers may be attributed to the marketability of poultry eggs over poultry meat, with eggs being affordable to the majority of the population [12]. Therefore, farmers may expect that it is more economically reliable to focus on layer production. Interestingly, egg consumption in Nigeria is higher than in many other African countries, likely due to poultry production being the most commercialised agricultural livestock sector in Nigeria. This contrasts with areas such as East Africa, which are dominated by smallholder farming [3,14]. Eggs and live spent layers were also ranked by farmers as two of the top three poultry products perceived to generate the highest income, additionally supporting the preference for layer production. By-products of poultry production, such as manure and used beddings, were also valued by farmers as a source of additional revenue. Other surveys have found that manure was a key product, often sold to crop farmers [11]. 

Though the most important products sold were quite similar between states, there were marked differences in the products perceived as less economically important. For instance, Oyo farmers more commonly sold live birds or whole fresh carcasses, whilst only Kano farmers reported selling chicken parts, such as heads, feet, and intestines. These differences may be explained by the North-South economic divide, with Northern states historically having lower economic growth and higher poverty compared to the Southern states [15]. Consequently, to accommodate the lower living standard, Kano farmers more often sell meat parts, as they are more affordable for consumers and, thus, easier to sell than a whole chicken. Parts such as meat cuts, guts, and gizzard were ranked very low in economic importance to the farmers, and therefore they contribute less to the overall revenue generated by the respondents.

### 4.2. Pharmaceutical Products 

#### 4.2.1. Antibiotics

The findings of this study showed that antibiotics were widely used on poultry farms in Oyo and Kano, with only one respondent reporting not administering antibiotics in the last production cycle. This supports previous work documenting the wide use of antibiotics in livestock production in Nigeria [16]. When farmers in the current study were asked about the problems they encountered when sourcing antibiotics, the unavailability of antibiotics and antibiotic ineffectiveness (antibiotics perceived not to work as expected) or poor quality (concentration of drug is perceived to be less than what is written on the label) were perceived as crucial issues. The reasons for the perceived antibiotic ineffectiveness or poor quality are unclear and were not investigated in the current study; however, the potential implications of this finding in relation to appropriate antimicrobial use and antimicrobial resistance (AMR) are important. It is possible that inappropriate use of an antibiotic may result in perceived ineffectiveness. In a previous study of animal health professionals in Nigeria, a high proportion of respondents recommended the use of antibiotics to manage non-bacterial pathogens, including viruses, protozoans, and even fungi [17]. Such indiscriminate use of antibiotics can exert selection pressure, allowing resistant strains to proliferate and lead to increased risk of AMR [18]. The choice and selection of antibiotics for use in poultry and other livestock can have implications on public health. Resistance to antibiotics that are frequently used in veterinary medicine (e.g., tetracyclines and beta-lactams) has been reported in livestock in Nigeria [19,20]. In the current study, over a third (42.9%) of the respondents reported using enrofloxacin in the previous production cycle. Fluoroquinolones are categorised as critically important antimicrobials (CIAs) for human health [21,22], and there is concern that the use of enrofloxacin in animals could lead to sharing of genes encoding resistance to fluoroquinolones in people [17]. 

Ensuring the prudent use of ‘last resort’ antimicrobials, such as enrofloxacin, by animal health professionals and farmers is necessary as part of efforts to tackle the problem of AMR in both animals and humans. The extent to which regulatory guidelines on antibiotic use were followed by veterinary professionals or poultry farmers, and the frequency of use of microbiological culture and antimicrobial sensitivity testing were not investigated in the current study. At the national level, the National Agency for Food and Drug Administration and Control (NAFDAC) is responsible for drug regulation in Nigeria. In addition, there is a Nigerian government action plan to tackle the threat of AMR [23]. Efforts of relevant entities including NAFDAC, government ministries (such as the Federal and State Ministries of Agriculture and Rural Development), private practices, and veterinary institutions will be critical in promoting the appropriate use of antibiotics by animal health professionals and farmers in Nigeria.

#### 4.2.2. Use of Medicines in Poultry Feed

In the present study, just under 40% of the study respondents provided medicated feed to their birds, of which toxin binders and minerals and vitamins were most often referenced. Drugs such as coccidiostats and growth promoters [24] can be easily and effectively administered to poultry flocks via feed. In contrast with other studies, in the current study, coccidiostats were not used very often (only by 12%). The administration of coccidiostats to young poultry has been demonstrated as an effective preventive measure against acute clinical coccidiosis [24]. Following ND, coccidiosis was the disease most frequently reported by poultry farmers recruited as part of this study (details of flock health have been reported by [9]); this finding suggests a low use of coccidiostats on the study farms. An enhanced promotion of appropriate use and distribution of coccidiostats in poultry could therefore be useful in reducing the coccidiosis-associated losses in productivity.

#### 4.2.3. Challenges 

Nearly half of the farmers had encountered challenges in obtaining pharmaceutical products (e.g., drugs, vaccines, and diagnostics) with antibiotic unavailability and distance from farm among the major problems. Both study states have large metropolitan hubs, Kano town in Kano state and Ibadan in Oyo state. It is likely that these locations are the main source for the vendors of veterinary drugs, feed, and supplies that are utilised by farmers, since most poultry farms, including those in this study, are located around the town fringes. Indeed, in the current study, most farmers reported that their source of pharmaceutical products was located in towns or cities. Distance to the source was very similar between the states (90% estimating less than 2 h away), suggesting that there are other factors underpinning the reported issues in availability and access to pharmaceutical products. 

Farmers from Kano consistently reported facing more challenges than Oyo farmers. A possible explanation for the observed differences in the challenges is that Kano is situated substantially further from the financial and industrial port—the city of Lagos. This means that the required poultry farm inputs (i.e., feed, pharmaceuticals) need to be transported longer distances and problems in the supply chains may have a bigger impact. In addition, as previously stated, the country retains its definitive north–south divide, in wealth, education, ethnicity, religion and politics. The amalgamation of these disparities is expected to result in more challenges, and such of higher magnitude, to be experienced by farmers in the northern states. For instance, it may be that there is a much lower number of potential vendors, including vet shops and pharmaceutical distributers, in Kano than in Oyo. It can also be expected that in northern states, the consequences of extrinsic challenges related to conflict, security and politics, may discourage establishment of vendors compared to the other regions of the country. 

On the other hand, it is also important to consider the farmers’ knowledge of the pharmaceutical products available to them. It may be that there are suitable and ample products available but poor promotion by pharmaceutical and agricultural companies or distributers lead to lack of farmer awareness of the products that can be sourced, particularly with regards to novel and alternative poultry pharmaceutical and feed options. 

#### 4.2.4. Access to Veterinary Services

A theme that consistently emerged in this study was the lack of use, or the lack of access to veterinary services (e.g., veterinarians, extension support officers, or veterinary laboratories). For example, on developing vaccination protocols, less than half consulted either a vet or other relevant animal health personnel; over a third of Oyo farmers did not consult or seek external guidance. It is commonplace for farmers to perform their own vaccinations, as was the case reported by most Kano farmers. The scarce referral to animal health professionals infers there may be insufficient sources of veterinary guidance and support available to poultry farmers in Nigeria. Lack of veterinary extension services can have negative effects on poultry management. For instance, in another study, the absence of veterinary support personnel was found to be significantly related to a lower rate of adoption of biosecurity measures by poultry farmers in northern Nigeria [25]. 

In the current study, the inadequate access to veterinary services was more pronounced in Kano compared to Oyo, which may be explained by a lower number of animal health professionals being available or recruited in the northern region of the country. This pattern may be driven by the education inequalities previously noted between the north and south, with southern states, such as Oyo, increasingly developing a more learned society and simultaneously, more opportunities for professional employment [26]. The low number of animal health professionals can also lead to poultry farmers turning to other sources for guidance which may be unreliable, such as consulting other farmers, unqualified pseudo animal health professionals, or relying on personal experience. This reinforces the crucial need for improving access to veterinary services, especially in northern states. Principally, this can be achieved by increasing opportunities for animal health education and veterinary trainings with support from local and national government sectors. Furthermore, provision of refresher trainings for animal health personnel will equip them with detailed current information to advise farmers. Moreover, enlisting community animal health support workers that are based at the local level will provide the access to veterinary guidance vitally required by rural poultry farmers. Cost was another important factor related to veterinary services which was reported as a limitation for farmers surveyed in the current study. This finding is in agreement with a prior survey, which reported challenges faced by poultry farmers in Oyo state as costs involved in obtaining animal health information, veterinary services constraints, and lack of extension agents [27].

The education level of farmers was not explored as part of this study; therefore, it is unknown as to what extent this may be linked to seeking and the use of veterinary services. For example, more educated farmers with a better grasp of science and animal welfare may be more knowledgeable regarding symptoms of disease, pharmaceutical use, and vaccination, which could mean that veterinary services are either more or less frequently accessed.

### 4.3. Study Limitations

This study has limitations that should be considered when interpreting the findings and their implications. An important limitation is the fact that data collection relied on self-reported questionnaires administered to farmers. As part of the responses were based on the farmer’s perceptions, available records and on recall of information, it is possible that there might have been recall bias leading to over- or under-reporting or misreporting of certain investigated parameters. The potential misreporting of information due to the respondent’s inclination to give answers that they perceive will be positively viewed by the study investigators (i.e., social desirability bias) is another potential limitation with self-reported surveys like the current one. Social desirability bias can result in under-reporting of ‘undesirable’ and over-reporting of ‘desirable’ behaviours [28]. This occurs often in qualitative studies and has been highlighted in previous surveys in farmers in Sub-Saharan Africa [29]. Additionally, questions related to challenges faced by respondents were asked as open questions, and responses to these questions were not explored further. Therefore, responses such as ‘poor vaccine quality’, ‘poor quality of drugs’ and ‘ineffective vaccines’ were the opinions of respondents and were not measured. 

## 5. Conclusions

Strategies on improving poultry productivity in Nigeria are recommended based on the findings from this study. An increased access to veterinary pharmaceuticals is required, particularly in Northern regions in Nigeria, accompanied by education of farmers and animal health professionals in the correct use of antibiotic products. There is a need for targeted measures to help farms access veterinary products, especially those required to treat and prevent conditions that are top priorities for farmers. This issue could be tackled by further investigating and then addressing problems in cross-country supply chains, by improving transport networks for movement of pharmaceutical products, and promoting establishment of vendors in northern states. Such efforts will depend on substantial financial investment and commitment from the major poultry-industry stakeholders, including pharmaceutical and agricultural businesses, relevant divisions of the local and national government, and individual vendors. 

Increase in access to essential veterinary services (e.g., animal health professionals, veterinary diagnostics laboratories, and community support officers) is needed. This may be achieved through the establishment of more animal health education programmes to increase relevant personnel in the field, providing refresher trainings, and deploying locally based veterinary support, particularly in northern and rural regions. Further investigation into the diagnostic services (i.e., veterinary laboratory services) available to farmers nationwide is also needed, because diagnostic support and preventive steps might result in effective antimicrobial use and a more sustainable poultry industry.

An increased administration of medicated feed to poultry to reduce disease burden, particularly coccidiostats, is recommended; this could be achieved by actively promoting and increasing awareness of the benefits of the medications. Furthermore, incorporating education on the administration of medicated feed into trainings targeted at poultry farmers, as well as animal health professionals would be of great use to ensure appropriate use and minimise the risk of development of AMR. Ensuring the development, production and distribution of effective medicated feed products nationwide would also help tackle this issue. Poultry associations at the national, state, and local levels can aid these efforts by disseminating educational resources on medicated feed to farmers, either through online means, published reports and factsheets, or through word-of-mouth by veterinary service providers. 

Finally, this study highlights the need for the establishment and implementation of nationwide standard quality control guidelines in new and existing hatcheries to ensure production of high-quality and healthy DOCs. 

## Figures and Tables

**Table 1:** Production information on broiler birds owned in previous production cycle.

Information on Broiler Birds	State	*n*	Mean	SD	Median	Min	Max
Average number of broiler birds reached slaughter weight	Total	13	546.3	871.4	200.0	35.0	2940.0
	Oyo	3	1637.3	1470.4	1929.0	43.0	2940.0
	Kano	10	219.0	127.5	200.0	35.0	450.0
Average slaughter age (weeks)	Total	16	6.8	1.3	6.5	5.0	10.0
	Oyo	4	7.0	2.0	6.0	6.0	10.0
	Kano	12	6.7	1.1	7.0	5.0	8.0
Average slaughter weight (kg)	Total	12	2.0	1.3	1.8	1.2	6.0
	Oyo	4	2.1	0.3	2.0	1.8	2.5
	Kano	8	2.0	1.6	1.4	1.2	6.0
Average price for a broiler bird at ready slaughter weight (NGN, USD)	Total (NGN)	16	1510.6	810.4	1300.0	530.0	3500.0
	(USD)		3.7	2.0	3.2	1.3	8.5
	Oyo (NGN)	6	1961.7	1201.1	1850.0	530.0	3500.0
	(USD)		4.8	2.9	4.5	1.3	8.5
	Kano (NGN)	10	1240.0	275.7	1300.0	800.0	1700.0
	(USD)		3.0	0.7	3.2	1.9	4.1
Average number of bird mortalities on farm	Total	16	380.6	888.8	50.0	7.0	2800.0
	Oyo	5	92.6	63.7	85.0	7.0	180.0
	Kano	11	511.6	1059.7	30.0	10.0	2800.0

Abbreviations: NGN, Nigerian Naira; USD: US dollar; SD, standard deviation. At the time of writing 1 US dollar = 411.400 NGN (August 2021).

**Table 2:** Production information on layer birds owned in previous production cycle.

Information on Layer Birds	State	*n*	Mean	SD	Median	Min	Max
Number of layer birds reaching maturity	Total	30	5958.6	11,255.7	2797.0	90.0	56,000.0
	Oyo	12	4694.0	4120.6	3210.0	160.0	11,800.0
	Kano	18	6801.7	14,256.6	992.5	90.0	56,000.0
Length of production cycle (weeks)	Total	31	78.7	22.2	80.0	20.0	130.0
	Oyo	13	88.5	12.7	88.0	63.0	114.0
	Kano	18	71.7	25.1	72.0	20.0	130.0
Average price per crate of eggs sold (NGN, USD)	Total (NGN)	32	783.4	98.2	800.0	400.0	900.0
	(USD)		1.9	0.2	1.9	1.0	2.2
	Oyo (NGN)	13	793.9	35.2	800.0	750.0	800.0
	(USD)		1.9	0.1	1.9	1.8	1.9
	Kano (NGN)	19	776.3	125.1	800.0	400.0	900.0
	(USD)		1.9	0.3	1.9	1.0	2.2
Number of spent-layer or off-layer birds sold for slaughter	Total	32	5381.4	9175.1	2440.0	80.0	51,000.0
	Oyo	13	5722.3	4668.2	3750.0	800.0	14,250.0
	Kano	19	5148.2	11,415.4	2100.0	80.0	51,000.0
Average price for spent-layer or off-layer birds sold for slaughter at end of cycle (NGN, USD)	Total (NGN)	33	1185.8	172.7	1200.0	800.0	1500.0
	(USD)		2.9	0.4	2.9	1.9	3.6
	Oyo (NGN)	13	1296.2	114.5	1250.0	1100.0	1500.0
	(USD)		3.2	0.3	3.0	2.7	3.6
	Kano (NGN)	20	1114.0	167.9	1150.0	800.0	1350.0
	(USD)		2.7	0.4	2.8	1.9	3.3
Average number of bird mortalities occurred	Total	32	848.1	1278.0	290.0	7.0	5000.0
	Oyo	13	696.9	1116.2	278.0	7.0	3900.0
	Kano	19	951.6	1398.0	340.0	10.0	5000.0

Abbreviations: NGN, Nigerian Naira; USD: US dollar; SD, standard deviation. At the time of writing 1 US dollar = 411.400 NGN (August 2021).

**Table 3:** Estimated farm management costs for the previous production cycle.

Estimated Costs (NGN,	State	*n*	Mean	SD	Median	Min	Max
USD)							
Feeding broiler birds	Total (NGN)	11	522,913.6	562,704.6	260,000.0	32,000.0	1,650,000.0
	(USD)		1271.1	1367.8	632.0	77.8	4010.7
	Oyo (NGN)	4	783,200.0	731,249.1	725,400.0	32,000.0	1,650,000.0
	(USD)		1903.8	1777.5	1763.3	77.8	4010.7
	Kano (NGN)	7	374,178.6	435,189.2	190,000.0	65,000.0	1,200,000.0
	(USD)		909.5	1057.8	461.8	158.0	2916.9
Feeding layer birds	Total (NGN)	11	9,570,499.5	14,360,347.2	4,270,500.0	1,379,000.0	49,275,000.0
	(USD)		23,263.5	34,906.4	10,380.5	3352.0	119,775.2
	Oyo (NGN)	6	7,085,950.0	7,495,837.0	5,180,850.0	1,664,000.0	21,600,000.0
	(USD)		17,224.2	18,220.5	12,593.4	4044.8	52,504.2
	Kano (NGN)	5	12,551,959.0	20,614,160.0	4,270,500.0	1,379,000.0	49,275,000.0
	(USD)		30,510.7	50,107.9	10,380.5	3352.0	119,775.2
Antibiotic treatment	Total (NGN)	15	328,228.7	537,096.0	75,000.0	30.0	1,680,000.0
	(USD)		797.8	1305.5	182.3	0.1	4083.7
	Oyo (NGN)	5	576,280.0	640,416.4	400,000.0	75,000.0	1,680,000.0
	(USD)		1400.8	1556.7	972.3	182.3	4083.7
	Kano (NGN)	10	204,203.0	463,873.2	30,000.0	30.0	1,500,000.0
	(USD)		496.4	1127.6	72.9	0.1	3646.1
Vaccination programme (vaccines, vet, material for vaccination)	Total (NGN)	21	180,565.2	254,765.2	56,000.0	3200.0	750,000.0
	(USD)		438.9	619.3	136.1	7.8	1823.1
	Oyo (NGN)	6	290,920.0	346,756.6	103,000.0	29,520.0	750,000.0
	(USD)		707.2	842.9	250.4	71.8	1823.1
	Kano (NGN)	15	136,423.3	206,087.5	44,650.0	3200.0	652,000.0
	(USD)		331.6	500.9	108.5	7.8	1584.8
Maintaining biosecurity practices/procedures	Total (NGN)	32	44,210.9	80,694.1	13,500.0	2000.0	364,000.0
	(USD)		107.5	196.1	32.8	4.9	884.8
	Oyo (NGN)	9	63,277.8	116,185.5	15,000.0	2500.0	364,000.0
	(USD)		153.8	282.4	36.5	6.1	884.8
	Kano (NGN)	23	36,750.0	63,715.7	10,250.0	2000.0	300,000.0
	(USD)		89.3	154.9	24.9	4.9	729.2
Litter management	Total (NGN)	27	605,259.3	2,139,247.0	20,000.0	2000.0	10,800,000.0
	(USD)		1471.2	5200.0	48.6	4.9	26,252.1
	Oyo (NGN)	11	1,438,091.0	3,257,041.0	80,000.0	10,000.0	10,800,000.0
	(USD)		3495.6	7917.1	194.5	24.3	26,252.1
	Kano (NGN)	16	32,687.5	42,903.1	12,000.0	2000.0	156,000.0
	(USD)		79.5	104.3	29.2	4.9	379.2
Staff wages and benefits (manager, farm workers, etc)	Total (NGN)	39	1,288,910.0	2,867,937.0	540,000.0	20,000.0	18,000,000.0
	(USD)		3133.0	6971.2	1312.6	48.6	43,753.5
	Oyo (NGN)	12	1,119,625.0	1,097,514.0	600,000.0	72,000.0	3,240,000.0
	(USD)		2721.5	2667.8	1458.5	175.0	7875.6
	Kano (NGN)	27	1,364,148.0	3,390,063.0	384,000.0	20,000.0	18,000,000.0
	(USD)		3315.9	8240.4	933.4	48.6	43,753.5

Abbreviations: NGN, Nigerian Naira; USD, US dollar; SD, standard deviation. At the time of writing 1 US dollar = 411.400 NGN (August 2021).

**Table 4:** Source of the pharmaceutical products used on farm.

Source of Pharmaceutical Products Used on Farm	Total Respondents (*n*%) (*n* = 43) ^1^	Respondents in Kano (*n*%) (*n* = 27) ^1^	Respondents in Oyo (*n*%) (*n* = 16) ^1^
Vet shops	36 (83.7)	25 (92.6)	11 (68.7)
Vet pharmaceutical distributors	8 (18.6)	4 (14.8)	4 (25.0)
Veterinarian	2 (4.7)	0	2 (12.5)
Other	0	0	0

^1^ Respondents selected all applicable sources; therefore, column percentages do not add up to 100%.

**Table 5:** Time taken to travel from farm to source of the pharmaceutical products.

Average Amount of Travel Time from Farm to Source (Vet-Shop, etc.) Where Pharmaceutical Products Are Routinely Purchased/Accessed	Total Respondents (*n*%) (*n* = 43) ^1^	Respondents in Kano (*n*%) (*n* = 27) ^1^	Respondents in Oyo (*n*%) (*n* = 16) ^1^
Less than 60 min	21 (48.8)	13 (48.2)	8 (50.0)
Between 1 and 2 h	18 (41.9)	12 (44.4)	6 (37.5)
Between 2 and 3 h	2 (4.7)	2 (7.4)	0
>3 h	1 (2.3)	0	1 (6.3)
Not known	1 (2.3)	0	1 (6.3)

^1^ Percentages in each column may not add up to 100% due to rounding up.

**Table 6:** Top challenges faced on farm in relation to the vaccination programme.

Challenge ^1^	Total Respondents (*n*%) (*n* = 42) ^2^	Respondents in Kano (*n*%) (*n* = 26)	Respondents in Oyo (*n*%) (*n* = 16)
No challenge reported	10 (23.4)	4 (15.4)	6 (37.5)
Health issues interfering with schedule	7 (16.7)	7 (26.9)	0
Unavailability of vaccines	6 (14.3)	6 (23.1)	0
Long distance from source	6 (14.3)	6 (23.1)	0
Poor vaccine quality	4 (9.5)	3 (11.5)	1 (6.3)
Storage issues (cold chain)	4 (9.5)	2 (7.7)	2 (12.5)
Unavailability of vet	4 (9.5)	3 (11.5)	1 (6.3)
Ineffective vaccines	3 (7.2)	2 (7.7)	1 (6.3)
Negative vaccine effects	2 (4.8)	0	2 (12.5)
Multi-age structure of farm	2 (4.8)	0	2 (12.5)
Difficult handling chickens	1 (2.4)	0	1 (6.3)
High vaccine costs	1 (2.4)	1 (3.8)	0
Vaccine small packages unavailability	1 (2.4)	1 (3.8)	0
Poor water quality affecting vaccineeffectiveness	1 (2.4)	0	1 (6.3)

^1^ Respondents selected all challenges applicable to them, therefore column percentages may not add up to 100%. ^2^ There was one missing value for the vaccination programme challenges variable; this was excluded (total = 42, instead of 43).

## Data Availability

Data is contained within the article or Supplementary Material.

## Supplementary Materials

The following are available online at https://www.mdpi.com/article/10.3390/vetsci8120315/s1, Table S1: Respondent demographics and general farm characteristics as presented in Ekiri et al., unpublished. Table S2: Average number of chicks at the beginning of the previous production cycle on farm (if applicable). Table S3: Average cost (NGN) of a one-day old chick hatched or purchased for raising on farm in the previous production/hatching cycle (if applicable). Table S4: Average number of chicks hatched at hatchery/hatcheries on-farm in the previous production cycle (if applicable). Table S5: Number of unproductive birds culled from farm during the previous production cycle. Table S6: Estimated cost of vaccines (NGN) per 1000 birds in the previous production cycle. Table S7: Antibiotics used on the farm during the previous production cycle. Table S8: Duration of antibiotic use during the previous production cycle. Table S9: Advice on vaccination protocols, source of vaccines purchased by respondents, and person performing the vaccination on farm. Questionnaire S1: Questionnaire.
