# Supplementary material for: Baseline Assessment of Poultry Production, Pharmaceutical Product Use, and Related Challenges on Commercial Poultry Flocks in Kano and Oyo States of Nigeria
Source: Vet Sci. 2021 Dec 8;8(12):315. doi: 10.3390/vetsci8120315 (PMC8709114; doi:10.3390/vetsci8120315)
Supplement: Supplementary file 1 [file vetsci-08-00315-s001.zip › supplementary/Questionnaire S1.pdf]

Questionnaire S1

Q2 Please choose the **unique FARM ID from the list** provided to you. Be careful to choose the correct farm ID.

____

Q3 Please confirm your **unique FARM ID** code

____

Q4 Date of farm visit:

	Month	Day	Year
Please Select: (1)	▼ January (1 ... December (12)	▼ 1 (1 ... 31 (31)	▼ 1900 (1 ... 2049 (150)

Q5 Please insert the "3 words" ID generated with what3word app:

____
____
____
____
____

Q6 In what language are you conducting the survey? Please select all that apply

- English (1)
- Yoruba (2)
- Igbo (3)
- Hausa (4)
- Other, please specify (5) ____

End of Block: Farm ID & location

Start of Block: Farmer demographic information

Q7 What are the names of the farm owner? (if applicable):

____

Q8 What is the age of the farm owner?

- Less than 35 years (1)
- 35 – 45 years (2)
- 46 – 60 years (3)
- Above 60 years (4)

Q9 What is the gender of the farm owner?

- Male (1)
- Female (2)

Q10 How many years of experience in poultry farming does the farm owner have?

- 0-5 years (1)
- 6-10 years (2)
- 11-20 years (3)
- Above 20 years (4)

Q11 What is the current role on the farm of the person completing the survey? Please check all that apply

- Farm owner (1)
- Farm manager (2)
- Farm supervisor (3)
- Farm worker (4)
- Farm vet (6)
- Other, please specify (5) ____

Q12 Please indicate if you/your farm are a member of the following farmer groups. Check all that apply:

- ☐ Poultry association of Nigeria at zonal/local government level (1)
- ☐ Poultry association of Nigeria at state level (2)
- ☐ Poultry association of Nigeria at national level (3)
- ☐ Other, please specify (4) ____

End of Block: Farmer demographic information

Start of Block: Farm information

Q13 What is the name of your farm (if applicable)?

____

____

____

____

____

Q14 Where is your farm located (complete where applicable)?

- ○ Village (1) ____
- ○ Town/city (2) ____
- ○ Local government area (5) ____
- ○ State (6) ____

Q15 What type of animals do you keep on your farm ? Please check all that apply.

- ▢ Cattle (1)
- ▢ Goats (2)
- ▢ Pigs (3)
- ▢ Sheep (4)
- ▢ Chickens (broilers or layers or breeders or free-ranging) (5)
- ▢ Ducks or geese (6)
- ▢ Turkeys (7)
- ▢ Rabbits (8)
- ▢ Other, please specify (9) ____

Q16 For how long has the farm been in existence (years)?

- ○ 0-5 years (1)
- ○ 6-10 years (2)
- ○ 11-20 years (3)
- ○ Above 20 years (4)

Q17 What **types of chickens** are kept on your farm at present (as of visit date)? (PS = parent stock, GPS = grand-parent stock). Please check all that apply.

- ▢ Layers (chicks or adult birds) (1)
- ▢ Broilers (chicks or adult birds) (2)
- ▢ Breeders-broiler (GPS chicks or adult birds) (3)
- ▢ Breeders-broiler (PS chicks or adult birds) (4)
- ▢ Breeders-layers (GPS chicks or adult birds) (5)
- ▢ Breeders-layers (PS chicks or adult birds) (6)
- ▢ Free-ranging chickens (7)
- ▢ Cockerels (8)
- ▢ Other type, please specify (9) ____

End of Block: Farm information

Start of Block: General management and house/shed information

Display This Question:

If What types of chickens are kept on your farm at present (as of visit date)? (PS = parent stock, G... = Layers (chicks or adult birds)

*

Q18 If you selected "Layers", please specify the **estimated number** of birds kept on your farm at present (as of visit date):

____

Display This Question:

If What types of chickens are kept on your farm at present (as of visit date)? (PS = parent stock, G... = Broilers (chicks or adult birds)

*

Q19 If you selected "Broilers", please specify the **estimated** number of birds kept on your farm at present (as of visit date):

____

Display This Question:

If What types of chickens are kept on your farm at present (as of visit date)? (PS = parent stock, G... = Breeders-broiler (GPS chicks or adult birds)

*

Q20 If you selected "GPS Breeders-broiler", please specify the **estimated** number of birds kept on your farm at present (as of visit date):

____

Display This Question:
If What types of chickens are kept on your farm at present (as of visit date)? (PS = parent stock, G... = Breeders-broiler (PS chicks or adult birds)

Q21 If you selected "PS Breeders-broiler", please specify the **estimated** number of birds kept on your farm at present (as of visit date):

____

Display This Question:
If What types of chickens are kept on your farm at present (as of visit date)? (PS = parent stock, G... = Breeders-layers (GPS chicks or adult birds)

Q22 If you selected "GPS Breeders-layers", please specify the **estimated** number of birds kept on your farm at present (as of visit date):

____

Display This Question:
If What types of chickens are kept on your farm at present (as of visit date)? (PS = parent stock, G... = Breeders-layers (PS chicks or adult birds)

Q23 If you selected "PS Breeders-layers", please specify the **estimated** number of birds kept on your farm at present (as of visit date):

____

Display This Question:
If What types of chickens are kept on your farm at present (as of visit date)? (PS = parent stock, G... = Other type, please specify

Q24 If you selected "Other", please specify the **estimated number** of birds kept on your farm at present (as of visit date):

____

Q25 What is the flock structure on your farm at present?

- ○ Single-age flock (1)
- ○ Multi-age flock (3)
- ○ Other, please specify (4) ________________________________

Q26 What is the type of rearing system on your farm at present? Please check all that apply

- ▢ Deep litter system (1)
- ▢ Battery cage system (2)
- ▢ Other, please specify (3) ________________________________

Q27 What type of housing system do you currently use on your farm? Please check all boxes that apply

- ☐ Closed House System (1)
- ☐ Open House System (2)
- ☐ Other, please specify (3) ____

Q28 With regards to physical structure, what best describes the housing structure currently used on your farm? Please check all the boxes that apply

- ☐ Brick, wood & wire mesh walls (1)
- ☐ Wood & wire mesh walls (2)
- ☐ Concrete floors (3)
- ☐ Wooden floors (5)
- ☐ Other, please specify (4) ____

---

*

Q29 How many bird **houses** do you currently have on your farm?

____

---

*

Q30 How many bird houses on your farm are **currently in use**?

____

---

*

Q31 Of the bird houses currently in use on your farm, what is the average number of birds **if known**?

____

End of Block: General management and house/shed information

Start of Block: Biosecurity, litter and vaccination information

Q32 Which of the following measures or practices do you currently have in place to minimise risk of spread of disease among birds on your farm? Please check all boxes that apply

	Never (1)	Sometimes (2)	Always (4)	Not known (3)
Bird house secured against access to neighbours' flocks, free-ranging chicken or ducks or geese or wild birds (264)	○	○	○	○
Practice "All-in and all-out" at end of production cycle (265)	○	○	○	○

Q33 Which of the following **traffic control** measures or practices do you currently have in place to minimise risk of spread of disease among birds on your farm? Please check all boxes that apply

	Never (4)	Sometimes (5)	Always (6)	Not known (7)
Keep a record of all visitors to your farm (79)	○	○	○	○
Bird house secured against access and contact with unauthorised personnel (80)	○	○	○	○
Minimise vehicles transit on the farm premises (81)	○	○	○	○

Q34 Which of the following **measures or practices** do you put in place to minimise risk of spread of disease among birds on your farm? Please check all boxes that apply

	Never (4)	Sometimes (5)	Always (6)	Not known (7)
Isolation/quarantine of infected birds (80)	○	○	○	○
Treating affected birds (81)	○	○	○	○
Culling of diseased birds (79)	○	○	○	○
Dead birds' removal from houses daily (82)	○	○	○	○
Dispose dead birds (83)	○	○	○	○

Q35 Which of the following measures or practices do you currently have in place to minimise risk of spread of disease among birds on your farm? Please check all boxes that apply

	Never (4)	Sometimes (5)	Always (6)	Not known (7)
Vaccination (216)	○	○	○	○
Wearing gloves when handling birds (199)	○	○	○	○
Washing hands with soap and water after handling birds (200)	○	○	○	○
Equipment cleaned and disinfected before being taken into a poultry house (201)	○	○	○	○
Changing footwear or wearing plastic boots before and after visiting bird house (202)	○	○	○	○
Working footbaths on the farm, located at farm entrance or outside the bird houses (203)	○	○	○	○
Change the litter between each batch of chickens or at end of each production cycle (205)	○	○	○	○
Clean out sheds/houses between each batch of chickens (cleaning may include washing and disinfection) (206)	○	○	○	○
Farm access routes, parking areas, yards, feeding and storage areas kept clean and tidy (207)	○	○	○	○
Have bird house structures (walls and floor materials) that can be easily cleaned using washing and disinfection (208)	○	○	○	○
Other (209)	○	○	○	○

Q36 How do you dispose of dead birds? Please check all that apply

- ☐ Buried (1)
- ☐ Burnt (2)
- ☐ Thrown away/binned (3)
- ☐ Sold off to ready buyers (4)
- ☐ Recycled into feed/composted and used in fertilizers (5)
- ☐ Other, please specify (6) ____

Q37 How do you dispose of the dead birds during a disease outbreak? Please check all that apply

- ☐ Buried (1)
- ☐ Burnt (2)
- ☐ Thrown away/binned (3)
- ☐ Other, please specify (4) ____

Q38 What type of litter system do you currently use on your farm? Please check all boxes that apply

- ☐ Wood shavings (1)
- ☐ Saw dust (2)
- ☐ Rice hulls (3)
- ☐ Peanut hulls (4)
- ☐ Straw (5)
- ☐ Other, please specify (6) ____

Q39 What factors influence the most the type of litter that you use on your farm? Please check all boxes that apply

- ▢ Price (1)
- ▢ Availability (2)
- ▢ Season (3)
- ▢ Other, please specify (4) ____

Use Reusable Choices

Q40 Did you vaccinate your birds during the previous production cycle?

- ◯ No (x1)
- ◯ Yes (x2)
- ◯ Not known (x3)
- ◯ Not applicable (x4)

Display This Question:

If If Did you vaccinate your birds during the previous production cycle? Yes Is Selected

Q41 For which of the diseases listed below did you vaccinate against in the previous production cycle? Please check all boxes that apply

	Breeder birds (1)	Broiler birds (2)	Layer birds (3)
Infectious bronchitis (1)	▢	▢	▢
Marek's disease (2)	▢	▢	▢
Newcastle disease (3)	▢	▢	▢
Gumboro (infectious bursal disease) (4)	▢	▢	▢
Avian encephalomyelitis (5)	▢	▢	▢
Egg drop syndrome (6)	▢	▢	▢
Fowl pox (7)	▢	▢	▢
Infectious laryngotrachitis (20)	▢	▢	▢
Tenosynovitis (8)	▢	▢	▢
Colibacillosis (Escherichia coli) (10)	▢	▢	▢
Fowl typhoid (salmonellosis) (16)	▢	▢	▢
Fowl cholera (pasteurellosis) (13)	▢	▢	▢
Infectious coryza (19)	▢	▢	▢
Coccidiosis (11)	▢	▢	▢
Chronic respiratory disease (CRD, Mycoplasmosis) (9)	▢	▢	▢
Other, please **specify the disease** (12)	▢	▢	▢

Display This Question:
If For which of the diseases listed below did you vaccinate against in the previous production cycle... = Infectious bronchitis [Breeder birds]
Or For which of the diseases listed below did you vaccinate against in the previous production cycle... = Infectious bronchitis [Broiler birds]
Or For which of the diseases listed below did you vaccinate against in the previous production cycle... = Infectious bronchitis [Layer birds]

Q42 If you selected infectious bronchitis , **select one or more methods** of vaccine application used on your farm in previous production cycle. Please check all boxes that apply

▢ Embryo Injection (1)

▢ Aerosol/droplets (2)

▢ Eye-drop (3)

▢ Water administration (4)

▢ Wing-web puncture (5)

▢ Feather follicle inoculation (6)

▢ Injection under the skin (7)

▢ Injection in muscle (8)

▢ Other (9)

▢ ⊗Not known (10)

Display This Question:
If For which of the diseases listed below did you vaccinate against in the previous production cycle... = Marek's disease [Breeder birds]
Or For which of the diseases listed below did you vaccinate against in the previous production cycle... = Marek's disease [Broiler birds]
Or For which of the diseases listed below did you vaccinate against in the previous production cycle... = Marek's disease [Layer birds]

Q43 If you selected <u>Marek's disease</u>, **select one or more methods** of vaccine application used on your farm in previous production cycle. Please check all boxes that apply

- ▢ Embryo Injection (1)
- ▢ Aerosol/droplets (2)
- ▢ Eye-drop (3)
- ▢ Water administration (4)
- ▢ Wing-web puncture (5)
- ▢ Feather follicle inoculation (6)
- ▢ Injection under the skin (7)
- ▢ Injection in muscle (8)
- ▢ Other (9)
- ▢ ⊗Not known (10)

Display This Question:
If For which of the diseases listed below did you vaccinate against in the previous production cycle... = Newcastle disease [Breeder birds]
Or For which of the diseases listed below did you vaccinate against in the previous production cycle... = Newcastle disease [Broiler birds]
Or For which of the diseases listed below did you vaccinate against in the previous production cycle... = Newcastle disease [Layer birds]

Q44 If you selected Newcastle disease, **select one or more methods** of vaccine application used on your farm in previous production cycle. Please check all boxes that apply

- ☐ Embryo Injection (1)
- ☐ Aerosol/droplets (2)
- ☐ Eye-drop (3)
- ☐ Water administration (4)
- ☐ Wing-web puncture (5)
- ☐ Feather follicle inoculation (6)
- ☐ Injection under the skin (7)
- ☐ Injection in muscle (8)
- ☐ Other (9)
- ☐ ⊗Not known (10)

Display This Question:

If For which of the diseases listed below did you vaccinate against in the previous production cycle... = Gumboro (infectious bursal disease) [Breeder birds]

Or For which of the diseases listed below did you vaccinate against in the previous production cycle... = Gumboro (infectious bursal disease) [Broiler birds]

Or For which of the diseases listed below did you vaccinate against in the previous production cycle... = Gumboro (infectious bursal disease) [Layer birds]

Q45 If you selected Gumboro (infectious bursal disease) , **select one or more methods** of vaccine application used on your farm in previous production cycle. Please check all boxes that apply

- ▢ Embryo Injection (1)
- ▢ Aerosol/droplets (2)
- ▢ Eye-drop (3)
- ▢ Water administration (4)
- ▢ Wing-web puncture (5)
- ▢ Feather follicle inoculation (6)
- ▢ Injection under the skin (7)
- ▢ Injection in muscle (8)
- ▢ Other (9)
- ▢ ⊗Not known (10)

Display This Question:

If For which of the diseases listed below did you vaccinate against in the previous production cycle... = Avian encephalomyelitis [Breeder birds]

Or For which of the diseases listed below did you vaccinate against in the previous production cycle... = Avian encephalomyelitis [Broiler birds]

Or For which of the diseases listed below did you vaccinate against in the previous production cycle... = Avian encephalomyelitis [Layer birds]

Q46 If you selected avian encephalomyelitis, **select one or more methods** of vaccine application used on your farm in the previous production cycle. Please check all boxes that apply

- ▢ Embryo Injection (1)
- ▢ Aerosol/droplets (2)
- ▢ Eye-drop (3)
- ▢ Water administration (4)
- ▢ Wing-web puncture (5)
- ▢ Feather follicle inoculation (6)
- ▢ Injection under the skin (7)
- ▢ Injection in muscle (8)
- ▢ Other (9)
- ▢ ⊗Not known (10)

Display This Question:

If For which of the diseases listed below did you vaccinate against in the previous production cycle... = Egg drop syndrome [Breeder birds]

Or For which of the diseases listed below did you vaccinate against in the previous production cycle... = Egg drop syndrome [Broiler birds]

Or For which of the diseases listed below did you vaccinate against in the previous production cycle... = Egg drop syndrome [Layer birds]

Q47 If you selected egg drop syndrome , **select one or more methods** of vaccine application used on your farm in previous production cycle. Please check all boxes that apply

- ▢ Embryo Injection (1)
- ▢ Aerosol/droplets (2)
- ▢ Eye-drop (3)
- ▢ Water administration (4)
- ▢ Wing-web puncture (5)
- ▢ Feather follicle inoculation (6)
- ▢ Injection under the skin (7)
- ▢ Injection in muscle (8)
- ▢ Other (9)
- ▢ ⊗Not known (10)

Display This Question:

If For which of the diseases listed below did you vaccinate against in the previous production cycle... = Fowl pox [Breeder birds]

Or For which of the diseases listed below did you vaccinate against in the previous production cycle... = Fowl pox [Broiler birds]

Or For which of the diseases listed below did you vaccinate against in the previous production cycle... = Fowl pox [Layer birds]

Q48 If you selected fowl pox, **select one or more methods** of vaccine application used on your farm in previous production cycle.
Please check all boxes that apply

- ☐ Embryo Injection (1)
- ☐ Aerosol/droplets (2)
- ☐ Eye-drop (3)
- ☐ Water administration (4)
- ☐ Wing-web puncture (5)
- ☐ Feather follicle inoculation (6)
- ☐ Injection under the skin (7)
- ☐ Injection in muscle (8)
- ☐ Other (9)
- ☐ ⊗Not known (10)

Display This Question:
If For which of the diseases listed below did you vaccinate against in the previous production cycle... = Infectious laryngotrachitis [Breeder birds]
Or For which of the diseases listed below did you vaccinate against in the previous production cycle... = Infectious laryngotrachitis [Broiler birds]
Or For which of the diseases listed below did you vaccinate against in the previous production cycle... = Infectious laryngotrachitis [Layer birds]

Q49 If you selected infectious laryngotrachitis, **select one or more methods** of vaccine application used on your farm in previous production cycle. Please check all boxes that apply

- ▢ Embryo Injection (1)
- ▢ Aerosol/droplets (2)
- ▢ Eye-drop (3)
- ▢ Water administration (4)
- ▢ Wing-web puncture (5)
- ▢ Feather follicle inoculation (6)
- ▢ Injection under the skin (7)
- ▢ Injection in muscle (8)
- ▢ Other (9)
- ▢ ⊗Not known (10)

Display This Question:

If For which of the diseases listed below did you vaccinate against in the previous production cycle... = Tenosynovitis [Breeder birds]

Or For which of the diseases listed below did you vaccinate against in the previous production cycle... = Tenosynovitis [Broiler birds]

Or For which of the diseases listed below did you vaccinate against in the previous production cycle... = Tenosynovitis [Layer birds]

Q50 If you selected tenosynovitis, **select one or more methods** of vaccine application used on your farm in previous production cycle. Please check all boxes that apply

- ▢ Embryo Injection (1)
- ▢ Aerosol/droplets (2)
- ▢ Eye-drop (3)
- ▢ Water administration (4)
- ▢ Wing-web puncture (5)
- ▢ Feather follicle inoculation (6)
- ▢ Injection under the skin (7)
- ▢ Injection in muscle (8)
- ▢ Other (9)
- ▢ ⊗Not known (10)

Display This Question:

If For which of the diseases listed below did you vaccinate against in the previous production cycle... = Colibacillosis (Escherichia coli) [Breeder birds]

Or For which of the diseases listed below did you vaccinate against in the previous production cycle... = Colibacillosis (Escherichia coli) [Broiler birds]

Or For which of the diseases listed below did you vaccinate against in the previous production cycle... = Colibacillosis (Escherichia coli) [Layer birds]

Q51 If you selected colibacillosis, **select one or more methods** of vaccine application used on your farm in previous production cycle. Please check all boxes that apply

- ▢ Embryo Injection (1)
- ▢ Aerosol/droplets (2)
- ▢ Eye-drop (3)
- ▢ Water administration (4)
- ▢ Wing-web puncture (5)
- ▢ Feather follicle inoculation (6)
- ▢ Injection under the skin (7)
- ▢ Injection in muscle (8)
- ▢ Other (9)
- ▢ ⊗Not known (10)

Display This Question:

If For which of the diseases listed below did you vaccinate against in the previous production cycle... = Fowl typhoid (salmonellosis) [Breeder birds]

Or For which of the diseases listed below did you vaccinate against in the previous production cycle... = Fowl typhoid (salmonellosis) [Layer birds]

Or For which of the diseases listed below did you vaccinate against in the previous production cycle... = Fowl typhoid (salmonellosis) [Broiler birds]

Q52 If you selected fowl typhoid, **select one or more methods** of vaccine application used on your farm in previous production cycle. Please check all boxes that apply

- ▢ Embryo Injection (1)
- ▢ Aerosol/droplets (2)
- ▢ Eye-drop (3)
- ▢ Water administration (4)
- ▢ Wing-web puncture (5)
- ▢ Feather follicle inoculation (6)
- ▢ Injection under the skin (7)
- ▢ Injection in muscle (8)
- ▢ Other (9)
- ▢ ⊗Not known (10)

Display This Question:
If For which of the diseases listed below did you vaccinate against in the previous production cycle... = Fowl cholera (pasteurellosis) [Breeder birds]
Or For which of the diseases listed below did you vaccinate against in the previous production cycle... = Fowl cholera (pasteurellosis) [Broiler birds]
Or For which of the diseases listed below did you vaccinate against in the previous production cycle... = Fowl cholera (pasteurellosis) [Layer birds]

Q53 If you selected fowl cholera, **select one or more methods** of vaccine application used on your farm in previous production cycle. Please check all boxes that apply

- ▢ Embryo Injection (1)
- ▢ Aerosol/droplets (2)
- ▢ Eye-drop (3)
- ▢ Water administration (4)
- ▢ Wing-web puncture (5)
- ▢ Feather follicle inoculation (6)
- ▢ Injection under the skin (7)
- ▢ Injection in muscle (8)
- ▢ Other (9)
- ▢ ⊗Not known (10)

Display This Question:

If For which of the diseases listed below did you vaccinate against in the previous production cycle... = Infectious coryza [Breeder birds]

Or For which of the diseases listed below did you vaccinate against in the previous production cycle... = Infectious coryza [Broiler birds]

Or For which of the diseases listed below did you vaccinate against in the previous production cycle... = Infectious coryza [Layer birds]

Q54 If you selected infectious coryza, **select one or more methods** of vaccine application used on your farm in previous production cycle. Please check all boxes that apply

- ▢ Embryo Injection (1)
- ▢ Aerosol/droplets (2)
- ▢ Eye-drop (3)
- ▢ Water administration (4)
- ▢ Wing-web puncture (5)
- ▢ Feather follicle inoculation (6)
- ▢ Injection under the skin (7)
- ▢ Injection in muscle (8)
- ▢ Other (9)
- ▢ ⊗Not known (10)

Display This Question:

If For which of the diseases listed below did you vaccinate against in the previous production cycle... = Coccidiosis [Breeder birds]

Or For which of the diseases listed below did you vaccinate against in the previous production cycle... = Coccidiosis [Broiler birds]

Or For which of the diseases listed below did you vaccinate against in the previous production cycle... = Coccidiosis [Layer birds]

Q55 If you selected coccidiosis, **select one or more methods** of vaccine application used on your farm in previous production cycle. Please check all boxes that apply

- ▢ Embryo Injection (1)
- ▢ Aerosol/droplets (2)
- ▢ Eye-drop (3)
- ▢ Water administration (4)
- ▢ Wing-web puncture (5)
- ▢ Feather follicle inoculation (6)
- ▢ Injection under the skin (7)
- ▢ Injection in muscle (8)
- ▢ Other (9)
- ▢ ⊗Not known (10)

Display This Question:

If For which of the diseases listed below did you vaccinate against in the previous production cycle... = Chronic respiratory disease (CRD, Mycoplasmosis) [Breeder birds]

Or For which of the diseases listed below did you vaccinate against in the previous production cycle... = Chronic respiratory disease (CRD, Mycoplasmosis) [Broiler birds]

Or For which of the diseases listed below did you vaccinate against in the previous production cycle... = Chronic respiratory disease (CRD, Mycoplasmosis) [Layer birds]

Q56 If you selected mycoplasmosis, **select one or more methods** of vaccine application used on your farm in previous production cycle. Please check all boxes that apply

- ☐ Embryo Injection (1)
- ☐ Aerosol/droplets (2)
- ☐ Eye-drop (3)
- ☐ Water administration (4)
- ☐ Wing-web puncture (5)
- ☐ Feather follicle inoculation (6)
- ☐ Injection under the skin (7)
- ☐ Injection in muscle (8)
- ☐ Other (9)
- ☐ ⊗Not known (10)

Display This Question:

If For which of the diseases listed below did you vaccinate against in the previous production cycle... = Other, please specify the disease [Breeder birds]

Or For which of the diseases listed below did you vaccinate against in the previous production cycle... = Other, please specify the disease [Broiler birds]

Or For which of the diseases listed below did you vaccinate against in the previous production cycle... = Other, please specify the disease [Layer birds]

Q57 If you selected other disease, **select one or more methods** of vaccine application used on your farm in previous production cycle. Please check all boxes that apply

- ☐ Embryo Injection (1)
- ☐ Aerosol/droplets (2)
- ☐ Eye-drop (3)
- ☐ Water administration (4)
- ☐ Wing-web puncture (5)
- ☐ Feather follicle inoculation (6)
- ☐ Injection under the skin (7)
- ☐ Injection in muscle (8)
- ☐ Other (9)
- ☐ ⊗Not known (10)

Display This Question:
If If Did you vaccinate your birds during the previous production cycle? Yes Is Selected

Q58 For **each disease or combination of diseases (if applicable)**, please indicate the estimate **vaccine cost (Naira)** per 1000 birds:

For which of the diseases listed below did you vaccinate against in the previous production cycle... = Marek's disease [Breeder birds]
Or For which of the diseases listed below did you vaccinate against in the previous production cycle... = Marek's disease [Broiler birds]
Or For which of the diseases listed below did you vaccinate against in the previous production cycle... = Marek's disease [Layer birds]

☐ Infectious bronchitis (1) ____

For which of the diseases listed below did you vaccinate against in the previous production cycle... = Marek's disease [Breeder birds]
Or For which of the diseases listed below did you vaccinate against in the previous production cycle... = Marek's disease [Broiler birds]
Or For which of the diseases listed below did you vaccinate against in the previous production cycle... = Marek's disease [Layer birds]

☐ Marek's disease (2) ____

For which of the diseases listed below did you vaccinate against in the previous production cycle... = Newcastle disease [Breeder birds]
Or For which of the diseases listed below did you vaccinate against in the previous production cycle... = Newcastle disease [Broiler birds]
Or For which of the diseases listed below did you vaccinate against in the previous production cycle... = Newcastle disease [Layer birds]

☐ Newcastle disease (3) ____

For which of the diseases listed below did you vaccinate against in the previous production cycle... = Newcastle disease [Breeder birds]
Or For which of the diseases listed below did you vaccinate against in the previous production cycle... = Newcastle disease [Broiler birds]
Or For which of the diseases listed below did you vaccinate against in the previous production cycle... = Newcastle disease [Layer birds]
Or For which of the diseases listed below did you vaccinate against in the previous production cycle... = Infectious bronchitis [Breeder birds]
Or For which of the diseases listed below did you vaccinate against in the previous production cycle... = Infectious bronchitis [Broiler birds]
Or For which of the diseases listed below did you vaccinate against in the previous production cycle... = Infectious bronchitis [Layer birds]

☐ Newcastle disease + infectious bronchitis (4) ____

For which of the diseases listed below did you vaccinate against in the previous production cycle... = Newcastle disease [Breeder birds]
Or For which of the diseases listed below did you vaccinate against in the previous production cycle... = Newcastle disease [Broiler birds]
Or For which of the diseases listed below did you vaccinate against in the previous production cycle... = Newcastle disease [Layer birds]
Or For which of the diseases listed below did you vaccinate against in the previous production cycle... = Egg drop syndrome [Breeder birds]
Or For which of the diseases listed below did you vaccinate against in the previous production cycle... = Egg drop syndrome [Broiler birds]
Or For which of the diseases listed below did you vaccinate against in the previous production cycle... = Egg drop syndrome [Layer birds]
Or For which of the diseases listed below did you vaccinate against in the previous production cycle... = Infectious bronchitis [Breeder birds]
Or For which of the diseases listed below did you vaccinate against in the previous production cycle... = Infectious bronchitis [Broiler birds]
Or For which of the diseases listed below did you vaccinate against in the previous production cycle... = Infectious bronchitis [Layer birds]

☐ Newcastle disease + egg drop syndrome + infectious bronchitis (5) ____

For which of the diseases listed below did you vaccinate against in the previous production cycle... = Newcastle disease [Breeder birds]
Or For which of the diseases listed below did you vaccinate against in the previous production cycle... = Newcastle disease [Broiler birds]

Or For which of the diseases listed below did you vaccinate against in the previous production cycle... = Newcastle disease [Layer birds]
Or For which of the diseases listed below did you vaccinate against in the previous production cycle... = Egg drop syndrome [Breeder birds]
Or For which of the diseases listed below did you vaccinate against in the previous production cycle... = Egg drop syndrome [Broiler birds]
Or For which of the diseases listed below did you vaccinate against in the previous production cycle... = Egg drop syndrome [Layer birds]

▢ Newcastle disease + egg drop syndrome (6) ____

For which of the diseases listed below did you vaccinate against in the previous production cycle... = Newcastle disease [Breeder birds]
Or For which of the diseases listed below did you vaccinate against in the previous production cycle... = Newcastle disease [Broiler birds]
Or For which of the diseases listed below did you vaccinate against in the previous production cycle... = Newcastle disease [Layer birds]
Or For which of the diseases listed below did you vaccinate against in the previous production cycle... = Infectious coryza [Breeder birds]
Or For which of the diseases listed below did you vaccinate against in the previous production cycle... = Infectious coryza [Broiler birds]
Or For which of the diseases listed below did you vaccinate against in the previous production cycle... = Infectious coryza [Layer birds]

▢ Newcastle disease + infectious coryza (7) ____

For which of the diseases listed below did you vaccinate against in the previous production cycle... = Gumboro (infectious bursal disease) [Breeder birds]
Or For which of the diseases listed below did you vaccinate against in the previous production cycle... = Gumboro (infectious bursal disease) [Broiler birds]
Or For which of the diseases listed below did you vaccinate against in the previous production cycle... = Gumboro (infectious bursal disease) [Layer birds]

▢ Gumboro (infectious bursal disease) (8) ____

For which of the diseases listed below did you vaccinate against in the previous production cycle... = Avian encephalomyelitis [Breeder birds]
Or For which of the diseases listed below did you vaccinate against in the previous production cycle... = Avian encephalomyelitis [Broiler birds]
Or For which of the diseases listed below did you vaccinate against in the previous production cycle... = Avian encephalomyelitis [Layer birds]

▢ Avian encephalomyelitis (9) ____

For which of the diseases listed below did you vaccinate against in the previous production cycle... = Egg drop syndrome [Breeder birds]
Or For which of the diseases listed below did you vaccinate against in the previous production cycle... = Egg drop syndrome [Broiler birds]
Or For which of the diseases listed below did you vaccinate against in the previous production cycle... = Egg drop syndrome [Layer birds]

▢ Egg drop syndrome (10) ____

For which of the diseases listed below did you vaccinate against in the previous production cycle... = Fowl pox [Breeder birds]
Or For which of the diseases listed below did you vaccinate against in the previous production cycle... = Fowl pox [Broiler birds]
Or For which of the diseases listed below did you vaccinate against in the previous production cycle... = Fowl pox [Layer birds]

▢ Fowl pox (11) ____

For which of the diseases listed below did you vaccinate against in the previous production cycle... = Fowl pox [Breeder birds]
Or For which of the diseases listed below did you vaccinate against in the previous production cycle... = Fowl pox [Broiler birds]
Or For which of the diseases listed below did you vaccinate against in the previous production cycle... = Fowl pox [Layer birds]
Or For which of the diseases listed below did you vaccinate against in the previous production cycle... = Avian encephalomyelitis [Breeder birds]
Or For which of the diseases listed below did you vaccinate against in the previous production cycle... = Avian encephalomyelitis [Broiler birds]
Or For which of the diseases listed below did you vaccinate against in the previous production cycle... = Avian encephalomyelitis [Layer birds]

☐ Fowl pox +avian encephalomyelitis (12) ____

For which of the diseases listed below did you vaccinate against in the previous production cycle... = Tenosynovitis [Breeder birds]
Or For which of the diseases listed below did you vaccinate against in the previous production cycle... = Tenosynovitis [Broiler birds]
Or For which of the diseases listed below did you vaccinate against in the previous production cycle... = Tenosynovitis [Layer birds]

☐ Tenosynovitis (13) ____

For which of the diseases listed below did you vaccinate against in the previous production cycle... = Colibacillosis (Escherichia coli) [Breeder birds]
Or For which of the diseases listed below did you vaccinate against in the previous production cycle... = Colibacillosis (Escherichia coli) [Broiler birds]
Or For which of the diseases listed below did you vaccinate against in the previous production cycle... = Colibacillosis (Escherichia coli) [Layer birds]

☐ Colibacillosis (Escherichia coli) (14) ____

For which of the diseases listed below did you vaccinate against in the previous production cycle... = Fowl cholera (pasteurellosis) [Breeder birds]
Or For which of the diseases listed below did you vaccinate against in the previous production cycle... = Fowl cholera (pasteurellosis) [Broiler birds]
Or For which of the diseases listed below did you vaccinate against in the previous production cycle... = Fowl cholera (pasteurellosis) [Layer birds]
Or For which of the diseases listed below did you vaccinate against in the previous production cycle... = Fowl typhoid (salmonellosis) [Breeder birds]
Or For which of the diseases listed below did you vaccinate against in the previous production cycle... = Fowl typhoid (salmonellosis) [Broiler birds]
Or For which of the diseases listed below did you vaccinate against in the previous production cycle... = Fowl typhoid (salmonellosis) [Layer birds]

☐ Fowl cholera (pasteurellosis) + fowl typhoid (salmonellosis) (15) ____

For which of the diseases listed below did you vaccinate against in the previous production cycle... = Chronic respiratory disease (CRD, Mycoplasmosis) [Breeder birds]
Or For which of the diseases listed below did you vaccinate against in the previous production cycle... = Chronic respiratory disease (CRD, Mycoplasmosis) [Broiler birds]
Or For which of the diseases listed below did you vaccinate against in the previous production cycle... = Chronic respiratory disease (CRD, Mycoplasmosis) [Layer birds]

☐ Mycoplasmosis (16) ____

For which of the diseases listed below did you vaccinate against in the previous production cycle... = Coccidiosis [Breeder birds]
Or For which of the diseases listed below did you vaccinate against in the previous production cycle... = Coccidiosis [Broiler birds]
Or For which of the diseases listed below did you vaccinate against in the previous production cycle... = Coccidiosis [Layer birds]

☐ Coccidiosis (17) ____

For which of the diseases listed below did you vaccinate against in the previous production cycle... = Other, please specify the disease [Breeder birds]
Or For which of the diseases listed below did you vaccinate against in the previous production cycle... = Other, please specify the disease [Broiler birds]
Or For which of the diseases listed below did you vaccinate against in the previous production cycle... = Other, please specify the disease [Layer birds]

☐ Other disease specified (18) ____

Q59 Who gives you advice on the vaccines regime/protocol used on your farm? Please check all boxes that apply

- ▢ Government official (vet or paravet or agriculture extension officer) (1)
- ▢ My veterinarian (2)
- ▢ Vet shop attendant (4)
- ▢ ⊗No one, I just know what to do (6)
- ▢ Other, please specify (7) ____

Q60 Where do you source the vaccines/who supplies your vaccines? Please check all boxes that apply

- ▢ Government provides (vet or paravet or agriculture extension officer) (1)
- ▢ Vet shop (3)
- ▢ Vet pharmaceutical distributor (4)
- ▢ Veterinarian (5)
- ▢ Other, please specify (7) ____

End of Block: Biosecurity, litter and vaccination information

Start of Block: General management: nutrition information

Q61 What were the different types of feed provided to birds on your farm in the previous production cycle? Please check all boxes that apply

- ☐ Broiler pre-starter feed (1)
- ☐ Broiler starter feed (2)
- ☐ Broiler finisher feed (3)
- ☐ Broiler grower feed (4)
- ☐ Layer chick feed (5)
- ☐ Layer grower feed (6)
- ☐ Layer pre-lay feed (7)
- ☐ Layer feed (8)
- ☐ Other, please specify (9) ____

Display This Question:
If What were the different types of feed provided to birds on your farm in the previous production c... = Broiler pre-starter feed

Q62 If you selected **broiler pre-starter feed**, what form of feed was used on your farm in the previous production cycle? Please check all boxes that apply

- ☐ Mash (1)
- ☐ Pellet (2)
- ☐ Crumbles (3)

Display This Question:
If What were the different types of feed provided to birds on your farm in the previous production c... = Broiler starter feed

Q63 If you selected **broiler starter feed**, what form of feed was used on your farm in the previous production cycle? Please check all boxes that apply

- ☐ Mash (1)
- ☐ Pellet (2)
- ☐ Crumbles (3)

Display This Question:
If What were the different types of feed provided to birds on your farm in the previous production c... = Broiler finisher feed

Q64 If you selected **broiler finisher feed**, what form of feed was used on your farm in the previous production cycle? Please check all boxes that apply

☐ Mash (1)

☐ Pellet (2)

☐ Crumbles (3)

Display This Question:

If What were the different types of feed provided to birds on your farm in the previous production c... = Broiler grower feed

Q65 If you selected **broiler grower feed**, what form of feed was used on your farm in the previous production cycle? Please check all boxes that apply

☐ Mash (1)

☐ Pellet (2)

☐ Crumbles (3)

Display This Question:

If What were the different types of feed provided to birds on your farm in the previous production c... = Layer chick feed

Q66 If you selected **layer chick feed**, what form of feed was used on your farm in the previous production cycle? Please check all boxes that apply

☐ Mash (1)

☐ Pellet (2)

☐ Crumbles (3)

Display This Question:

If What were the different types of feed provided to birds on your farm in the previous production c... = Layer grower feed

Q67 If you selected **layer grower feed**, what form of feed was used on your farm in the previous production cycle? Please check all boxes that apply

☐ Mash (1)

☐ Pellet (2)

☐ Crumbles (3)

Display This Question:

If What were the different types of feed provided to birds on your farm in the previous production c... = Layer pre-lay feed

Q68 If you selected **layer pre-lay feed**, what form of feed was used on your farm in the previous production cycle? Please check all boxes that apply

- ☐ Mash (1)
- ☐ Pellet (2)
- ☐ Crumbles (3)

Display This Question:
If What were the different types of feed provided to birds on your farm in the previous production c... = Layer feed

Q69 If you selected **layer feed**, what form of feed was used on your farm in the previous production cycle? Please check all boxes that apply

- ☐ Mash (1)
- ☐ Pellet (2)
- ☐ Crumbles (3)

X→

Q70 **Where** did you buy or source **the feed** given to birds on your farm during the previous production cycle? Please check all boxes that apply

- ☐ Commercial feed mill (finished feed) (1)
- ☐ Self-mixed or self-prepared on farm feed mill (2)
- ☐ Feed made locally - self-mixing/feed rations prepared manually at home or on farm (3)
- ☐ Other, please specify (6) ____

Display This Question:
If Where did you buy or source the feed given to birds on your farm during the previous production c... = Self-mixed or self-prepared on farm feed mill
Or Where did you buy or source the feed given to birds on your farm during the previous production c... = Feed made locally - self-mixing/feed rations prepared manually at home or on farm

Q71 **Where** do you **buy the ingredients** for the feed that you mix or prepare at home? Please check all boxes that apply

- ☐ Commercial feedmill (2)
- ☐ Farm grown (3)
- ☐ Local supplier/Market (4)
- ☐ Others, please specify (5) ____

Display This Question:

If Where did you buy or source the feed given to birds on your farm during the previous production c... = Self-mixed or self-prepared on farm feed mill

Or Where did you buy or source the feed given to birds on your farm during the previous production c... = Feed made locally - self-mixing/feed rations prepared manually at home or on farm

Q72 What **ingredients** do you use in the self mixed or prepared feed? Please check all boxes that apply

▢ Soy meal (toasted or full fat) (1)

▢ Groundnut cake (2)

▢ Oyster shell (3)

▢ Maize (corn) bran (4)

▢ Rye and bran (5)

▢ Palm kernel cake (PKC) (6)

▢ Wheat Offal (7)

▢ Bone meal (8)

▢ Fish meal (9)

▢ Limestone (10)

▢ Additives - Methioline (11)

▢ Additives - Lysine (12)

▢ Additives - Salt (13)

▢ Other, please specify (14) ____

Q73 What is the key factor influencing the choice of feed purchased for your birds? Please check all boxes that apply

- ▢ Price (1)
- ▢ Quality of ingredients (evenly mixed feed, composition of feed) (2)
- ▢ Form of feed (mash, pellet, crumbles) (3)
- ▢ Distance from feed source (5)
- ▢ Availability (6)
- ▢ Other, please specify (7) ____

Use Reusable Choices

Q74 Did you provide feed mixed with medication (medicated feed) to your birds during the previous cycle?

- ○ No (x1)
- ○ Yes (x2)
- ○ Not known (x3)
- ○ Not applicable (x4)

Display This Question:

If If Did you provide feed mixed with medication (medicated feed) to your birds during the previous cycle? Yes Is Selected

Q75 What **type of medication** did you add to the feed? Please check all boxes that apply

- ▢ Antibiotics (1)
- ▢ Antifungals (2)
- ▢ Coccidiostats (3)
- ▢ Toxin binders (4)
- ▢ Lipolytic agents (5)
- ▢ Mineral and vitamins (6)
- ▢ Gut acidifiers (7)
- ▢ Probiotics (8)
- ▢ Growth promoters (9)
- ▢ ⊗Do not know (11)
- ▢ Other, please specify (10) ____

Display This Question:
If If Did you provide feed mixed with medication (medicated feed) to your birds during the previous cycle? Yes Is Selected

Q76 For each selected type of medication, please indicate to what **bird stage** the medicated feed was given in the previous production cycle. Please check all boxes that apply

	Broiler starter (1)	Broiler grower (2)	Broiler finisher (3)	Layer chick feed (4)	Layer grower feed (5)	Pre-lay feed (6)	Layer feed (7)
What type of medication did you add to the feed? Please check all boxes that apply = Antibiotics Antibiotics (1)	☐	☐	☐	☐	☐	☐	☐
What type of medication did you add to the feed? Please check all boxes that apply = Antifungals Antifungals (2)	☐	☐	☐	☐	☐	☐	☐
What type of medication did you add to the feed? Please check all boxes that apply = Coccidiostats Coccidiostats (3)	☐	☐	☐	☐	☐	☐	☐
What type of medication did you add to the feed? Please check all boxes that apply = Toxin binders Toxin binders (4)	☐	☐	☐	☐	☐	☐	☐
What type of medication did you add to the feed? Please check all boxes that apply = Lipolytic agents Lipolytic agents (5)	☐	☐	☐	☐	☐	☐	☐
What type of medication did you add to the feed? Please check all boxes that apply = Mineral and vitamins Mineral and vitamins (6)	☐	☐	☐	☐	☐	☐	☐

What type of medication did you add to the feed? Please check all boxes that apply = Gut acidifiers Gut acidifiers (7)	▢	▢	▢	▢	▢	▢	▢
What type of medication did you add to the feed? Please check all boxes that apply = Probiotics Probiotics (8)	▢	▢	▢	▢	▢	▢	▢
What type of medication did you add to the feed? Please check all boxes that apply = Growth promoters Growth promoters (9)	▢	▢	▢	▢	▢	▢	▢
What type of medication did you add to the feed? Please check all boxes that apply = Do not know ⊗Do not know (11)	▢	▢	▢	▢	▢	▢	▢
What type of medication did you add to the feed? Please check all boxes that apply = Other, please specify Other medication selected (10)	▢	▢	▢	▢	▢	▢	▢

Display This Question:

If What type of medication did you add to the feed? Please check all boxes that apply = Antibiotics

Q77 If you selected **antibiotics**, what commercial product did you use in the previous production cycle? Please check all boxes that apply

- ☐ Zinc Bacitracin (1)
- ☐ BMD (2)
- ☐ Oxynil Rx Dry (3)
- ☐ Aurofac 100g (4)
- ☐ Other, please specify (5) ____

Display This Question:
If What type of medication did you add to the feed? Please check all boxes that apply = Coccidiostats

Q78 If you selected **coccidiostats**, what commercial product did you use in the previous production cycle? Please check all boxes that apply

- ☐ Avatec (1)
- ☐ Cycostat (2)
- ☐ Cygro (3)
- ☐ Other, please specify (4) ____

End of Block: General management: nutrition information

Start of Block: Production information

X→

Q79 Do you have a hatchery on your farm or in another location?

- ◯ No (1)
- ◯ Yes (2)

Display This Question:
If Do you have a hatchery on your farm or in another location? = Yes

Q80 If farm has a hatchery, what is the type of eggs hatched in your hatchery or hatcheries? Please check all boxes that apply

- ☐ GPS eggs (1)
- ☐ PS eggs (2)
- ☐ Other, please specify (3) ____

Display This Question:
If Do you have a hatchery on your farm or in another location? = Yes

Q81 If farm has a hatchery, what is the source of the eggs used in the hatchery? Please check all boxes that apply

- ▢ Same farm (1)
- ▢ Commercial PS egg producer – broiler (2)
- ▢ Commercial PS egg producer – pullets/layers (3)
- ▢ Other, please specify (4) ____

Display This Question:
If Do you have a hatchery on your farm or in another location? = Yes

Q82 If farm has a hatchery, what is the type of chicks hatched at the hatchery in the previous production cycle? Please check all boxes that apply

- ▢ PS chicks (1)
- ▢ Broiler chicks (2)
- ▢ Pullet chicks (3)
- ▢ Cockerel chicks (5)
- ▢ Other, please specify (4) ____

Display This Question:
If Do you have a hatchery on your farm or in another location? = Yes

Q83 If farm has a hatchery, what is the average number of chicks produced per hatching?

____

Display This Question:
If Do you have a hatchery on your farm or in another location? = Yes

Q84 If farm has a hatchery, what is the final destination for the chicks from the hatcheries ? Please check all boxes that apply

- ▢ Grown on farm to adult size (as breeders, broilers or layers) (1)
- ▢ Sold to other farms (2)
- ▢ Sold to other companies running hatcheries (3)
- ▢ Other, please specify (4) ____

Display This Question:
If Do you have a hatchery on your farm or in another location? = No

Q85 If farm has no hatchery, what is the source of the broiler/layer day-old chicks? Please check all boxes that apply

- ▢ Commercial producer – broiler/layer chicks (1)
- ▢ Another small farm – broiler/layer chicks (2)
- ▢ Other, please specify (3) ____

Q86 Indicate the products obtained from chickens on your farm that are sold to generate money. Please check all boxes that apply

- ☐ Parent stock chicks (1)
- ☐ Broiler chicks (2)
- ☐ Pullet chicks (3)
- ☐ Cockerel chicks (4)
- ☐ Egg (PS eggs) (5)
- ☐ Egg (pullet/layer eggs) (6)
- ☐ Spent layers sold – live birds (7)
- ☐ Meat (ready weight broilers – live birds) (8)
- ☐ Whole fresh carcass (9)
- ☐ Meat cuts (10)
- ☐ Gizzard (11)
- ☐ Intestine/guts (12)
- ☐ Heads and feet (13)
- ☐ Used beddings (14)
- ☐ Feed (from farm feed mill or processed on farm by farmer) (15)
- ☐ Point of cage pullets (at 12-14 weeks of age) (16)
- ☐ Point of lay pullets (at 16 weeks of age) (17)
- ☐ Other, please specify (18) ____

Q87 Of the products checked above, indicate the **top** money-generating products for your farm. Please check all that apply [**Rank** the products in order of preference. Most preferred item at the top.]

Indicate the products obtained from chickens on your farm that are sold to generate money. Please... = Parent stock chicks

______ Parent stock chicks (1)

Indicate the products obtained from chickens on your farm that are sold to generate money. Please... = Broiler chicks

______ Broiler chicks (2)

Indicate the products obtained from chickens on your farm that are sold to generate money. Please... = Pullet chicks

______ Pullet chicks (3)

Indicate the products obtained from chickens on your farm that are sold to generate money. Please... = Cockerel chicks

______ Cockerel chicks (4)

Indicate the products obtained from chickens on your farm that are sold to generate money. Please... = Egg (PS eggs)

______ Egg (PS eggs) (5)

Indicate the products obtained from chickens on your farm that are sold to generate money. Please... = Egg (pullet/layer eggs)

______ Egg (pullet/layer eggs) (6)

Indicate the products obtained from chickens on your farm that are sold to generate money. Please... = Spent layers sold – live birds

______ Spent layers – live birds (7)

Indicate the products obtained from chickens on your farm that are sold to generate money. Please... = Meat (ready weight broilers – live birds)

______ Meat (ready weight broilers – live birds) (8)

Indicate the products obtained from chickens on your farm that are sold to generate money. Please... = Whole fresh carcass

______ Whole fresh carcass (9)

Indicate the products obtained from chickens on your farm that are sold to generate money. Please... = Meat cuts

______ Meat cuts (10)

Indicate the products obtained from chickens on your farm that are sold to generate money. Please... = Gizzard

______ Gizzard (11)

Indicate the products obtained from chickens on your farm that are sold to generate money. Please... = Intestine/guts

______ Intestine/guts (12)

Indicate the products obtained from chickens on your farm that are sold to generate money. Please... = Heads and feet

______ Heads and feet (13)

Indicate the products obtained from chickens on your farm that are sold to generate money. Please... = Used beddings

______ Litter/used beddings (14)

Indicate the products obtained from chickens on your farm that are sold to generate money. Please... = Feed (from farm feed mill or processed on farm by farmer)

______ Feed (from farm feed mill or processed on farm by farmer) (15)

Indicate the products obtained from chickens on your farm that are sold to generate money. Please... = Point of cage pullets (at 12-14 weeks of age)

______ Point of cage pullets (at 12-14 weeks of age) (16)

Indicate the products obtained from chickens on your farm that are sold to generate money. Please... = Point of lay pullets (at 16 weeks of age)

______ Point of lay pullets (at 16 weeks of age) (17)

Indicate the products obtained from chickens on your farm that are sold to generate money. Please... = Other, please specify

______ Other product (18)

X→

Q88 Indicate if these activities take place on your farm/premises. Please check all boxes that apply

- ▢ Operate a commercial feed mill (1)
- ▢ Operate a private feed mill (produce feed for farm use only) (2)
- ▢ Operate a slaughter house (3)
- ▢ Operate a slaughter house and processing plant (4)
- ▢ Processing of farm waste (beddings & litter) into manure for crops (5)
- ▢ Other, please specify (6) ____

End of Block: Production information

Start of Block: Flock health information

Q89 How often do you see the diseases/conditions mentioned below on your farm? **Please provide an answer for each disease/condition listed below**.

	Never (1)	Sometimes (2)	Frequently (3)	Not sure or not known (4)
Avian infectious bronchitis (868)	○	○	○	○
Marek's disease (869)	○	○	○	○
Avian influenza (870)	○	○	○	○
Newcastle disease (871)	○	○	○	○
Gumboro (infectious bursal disease) (872)	○	○	○	○
Avian encephalomyelitis (873)	○	○	○	○
Fowl pox (874)	○	○	○	○
Egg drop syndrome (875)	○	○	○	○
Escherichia coli (colibacillosis) (877)	○	○	○	○
Infectious coryza (878)	○	○	○	○
Necrotic Enteritis (879)	○	○	○	○
Fowl cholera (avian pasteurellosis) (880)	○	○	○	○
Fowl typhoid (salmonellosis) (889)	○	○	○	○
Chronic respiratory diseases (CRD, mycoplasmosis) (881)	○	○	○	○
Coccidiosis (882)	○	○	○	○
Worms (Ascaridia, tapeworms) (884)	○	○	○	○
Lice infestation (885)	○	○	○	○
Mycotoxins (Aflatoxicosis) (890)	○	○	○	○
Nutritional deficiencies (886)	○	○	○	○

Egg bound syndrome (887)	○	○	○	○
Other disease/condition (888)	○	○	○	○

Display This Question:
If How often do you see the diseases/conditions mentioned below on your farm? Please provide an answ... = Other disease/condition [Sometimes]
Or How often do you see the diseases/conditions mentioned below on your farm? Please provide an answ... = Other disease/condition [Frequently]

Q90 If you selected "other disease/condition", please specify which one/s you see on your farm.

____
____
____
____
____

Display This Question:
If How often do you see the diseases/conditions mentioned below on your farm? Please provide an answ... = Frequently

X→

Q91 Of the diseases or conditions selected, which of these have the **biggest impact** on your poultry **farm monetary profits**? [Rank the diseases in order of economic impact. Start with highest impact at the top.]

How often do you see the diseases/conditions mentioned below on your farm? Please provide an answ... = Avian infectious bronchitis [Frequently]

______ Avian infectious bronchitis (1)

How often do you see the diseases/conditions mentioned below on your farm? Please provide an answ... = Marek's disease [Frequently]

______ Marek's disease (2)

How often do you see the diseases/conditions mentioned below on your farm? Please provide an answ... = Avian influenza [Frequently]

______ Avian influenza (3)

How often do you see the diseases/conditions mentioned below on your farm? Please provide an answ... = Newcastle disease [Frequently]

______ Newcastle disease (4)

How often do you see the diseases/conditions mentioned below on your farm? Please provide an answ... = Gumboro (infectious bursal disease) [Frequently]

______ Gumboro (infectious bursal disease) (5)

How often do you see the diseases/conditions mentioned below on your farm? Please provide an answ... = Avian influenza [Frequently]

______ Avian encephalomyelitis (6)

How often do you see the diseases/conditions mentioned below on your farm? Please provide an answ... = Fowl pox [Frequently]

______ Fowl pox (7)

How often do you see the diseases/conditions mentioned below on your farm? Please provide an answ... = Egg drop syndrome [Frequently]

______ Egg drop syndrome (8)

How often do you see the diseases/conditions mentioned below on your farm? Please provide an answ... = Escherichia coli (colibacillosis) [Frequently]

______ Escherichia coli (colibacillosis) (10)

How often do you see the diseases/conditions mentioned below on your farm? Please provide an answ... = Infectious coryza [Frequently]

______ Infectious coryza (11)

How often do you see the diseases/conditions mentioned below on your farm? Please provide an answ... = Necrotic Enteritis [Frequently]

______ Necrotic enteritis (12)

How often do you see the diseases/conditions mentioned below on your farm? Please provide an answ... = Fowl cholera (avian pasteurellosis) [Frequently]

______ Fowl cholera (avian pasteurellosis) (13)

How often do you see the diseases/conditions mentioned below on your farm? Please provide an answ... = Chronic respiratory diseases (CRD, mycoplasmosis) [Frequently]

______ Chronic respiratory diseases (CRD, mycoplasmosis) (14)

How often do you see the diseases/conditions mentioned below on your farm? Please provide an answ... = Coccidiosis [Frequently]

______ Coccidiosis (15)

How often do you see the diseases/conditions mentioned below on your farm? Please provide an answ... = Worms (Ascaridia, tapeworms) [Frequently]

______ Worms (Ascaridia, tapeworms) (17)

How often do you see the diseases/conditions mentioned below on your farm? Please provide an answ... = Lice infestation [Frequently]

______ Lice infestation (18)

How often do you see the diseases/conditions mentioned below on your farm? Please provide an answ... = Mycotoxins (Aflatoxicosis) [Frequently]

______ Mycotoxicosis (Aflatoxicosis) (19)

How often do you see the diseases/conditions mentioned below on your farm? Please provide an answ... = Nutritional deficiencies [Frequently]

______ Nutritional deficiencies (20)

How often do you see the diseases/conditions mentioned below on your farm? Please provide an answ... = Egg bound syndrome [Frequently]

______ Egg bound syndrome (21)

How often do you see the diseases/conditions mentioned below on your farm? Please provide an answ... = Other disease/condition [Frequently]

______ Other disease/condition on your farm (22)

Display This Question:

If How often do you see the diseases/conditions mentioned below on your farm? Please provide an answ... = Frequently

Q92 Of the diseases or conditions selected, which of these have the **biggest impact** on productivity of your birds (e.g egg production, weight gain/loss, number of chicks, etc)? [Rank the diseases in order of impact on productivity. Start with highest impact at the top.]

How often do you see the diseases/conditions mentioned below on your farm? Please provide an answ... = Avian infectious bronchitis [Frequently]

______ Avian infectious bronchitis (1)

How often do you see the diseases/conditions mentioned below on your farm? Please provide an answ... = Marek's disease [Frequently]

______ Marek's disease (2)

How often do you see the diseases/conditions mentioned below on your farm? Please provide an answ... = Avian influenza [Frequently]

______ Avian influenza (3)

How often do you see the diseases/conditions mentioned below on your farm? Please provide an answ... = Newcastle disease [Frequently]

______ Newcastle disease (4)

How often do you see the diseases/conditions mentioned below on your farm? Please provide an answ... = Gumboro (infectious bursal disease) [Frequently]

______ Gumboro (infectious bursal disease) (5)

How often do you see the diseases/conditions mentioned below on your farm? Please provide an answ... = Avian influenza [Frequently]

______ Avian encephalomyelitis (6)

How often do you see the diseases/conditions mentioned below on your farm? Please provide an answ... = Fowl pox [Frequently]

______ Fowl pox (7)

How often do you see the diseases/conditions mentioned below on your farm? Please provide an answ... = Egg drop syndrome [Frequently]

______ Egg drop syndrome (8)

How often do you see the diseases/conditions mentioned below on your farm? Please provide an answ... = Escherichia coli (colibacillosis) [Frequently]

______ Escherichia coli (colibacillosis) (10)

How often do you see the diseases/conditions mentioned below on your farm? Please provide an answ... = Infectious coryza [Frequently]

______ Infectious coryza (11)

How often do you see the diseases/conditions mentioned below on your farm? Please provide an answ... = Necrotic Enteritis [Frequently]

______ Necrotic enteritis (12)

How often do you see the diseases/conditions mentioned below on your farm? Please provide an answ... = Fowl cholera (avian pasteurellosis) [Frequently]

______ Fowl cholera (avian pasteurellosis) (13)

How often do you see the diseases/conditions mentioned below on your farm? Please provide an answ... = Chronic respiratory diseases (CRD, mycoplasmosis) [Frequently]

______ Chronic respiratory diseases (CRD, mycoplasmosis) (14)

How often do you see the diseases/conditions mentioned below on your farm? Please provide an answ... = Coccidiosis [Frequently]

______ Coccidiosis (15)

How often do you see the diseases/conditions mentioned below on your farm? Please provide an answ... = Worms (Ascaridia, tapeworms) [Frequently]

______ Worms (Ascaridia, tapeworms) (17)

How often do you see the diseases/conditions mentioned below on your farm? Please provide an answ... = Lice infestation [Frequently]

______ Lice infestation (18)

How often do you see the diseases/conditions mentioned below on your farm? Please provide an answ... = Mycotoxins (Aflatoxicosis) [Frequently]

______ Mycotoxicosis (Aflatoxicosis) (19)

How often do you see the diseases/conditions mentioned below on your farm? Please provide an answ... = Nutritional deficiencies [Frequently]

______ Nutritional deficiencies (20)

How often do you see the diseases/conditions mentioned below on your farm? Please provide an answ... = Egg bound syndrome [Frequently]

______ Egg bound syndrome (21)

How often do you see the diseases/conditions mentioned below on your farm? Please provide an answ... = Other disease/condition [Frequently]

______ Other disease/condition on your farm (22)

Display This Question:

If How often do you see the diseases/conditions mentioned below on your farm? Please provide an answ... = Frequently

Q93 What diseases or conditions **cause the highest mortality** in birds on your farm (if known)? [Rank the diseases in order of relevance. Start with highest mortality at the top.]

How often do you see the diseases/conditions mentioned below on your farm? Please provide an answ... = Avian infectious bronchitis [Frequently]

_______ Avian infectious bronchitis (1)

How often do you see the diseases/conditions mentioned below on your farm? Please provide an answ... = Marek's disease [Frequently]

_______ Marek's disease (2)

How often do you see the diseases/conditions mentioned below on your farm? Please provide an answ... = Avian influenza [Frequently]

_______ Avian influenza (3)

How often do you see the diseases/conditions mentioned below on your farm? Please provide an answ... = Newcastle disease [Frequently]

_______ Newcastle disease (4)

How often do you see the diseases/conditions mentioned below on your farm? Please provide an answ... = Gumboro (infectious bursal disease) [Frequently]

_______ Gumboro (infectious bursal disease) (5)

How often do you see the diseases/conditions mentioned below on your farm? Please provide an answ... = Avian influenza [Frequently]

_______ Avian encephalomyelitis (6)

How often do you see the diseases/conditions mentioned below on your farm? Please provide an answ... = Fowl pox [Frequently]

_______ Fowl pox (7)

How often do you see the diseases/conditions mentioned below on your farm? Please provide an answ... = Egg drop syndrome [Frequently]

_______ Egg drop syndrome (8)

How often do you see the diseases/conditions mentioned below on your farm? Please provide an answ... = Escherichia coli (colibacillosis) [Frequently]

_______ Escherichia coli (colibacillosis) (10)

How often do you see the diseases/conditions mentioned below on your farm? Please provide an answ... = Infectious coryza [Frequently]

_______ Infectious coryza (11)

How often do you see the diseases/conditions mentioned below on your farm? Please provide an answ... = Necrotic Enteritis [Frequently]

_______ Necrotic enteritis (12)

How often do you see the diseases/conditions mentioned below on your farm? Please provide an answ... = Fowl cholera (avian pasteurellosis) [Frequently]

_______ Fowl cholera (avian pasteurellosis) (13)

How often do you see the diseases/conditions mentioned below on your farm? Please provide an answ... = Chronic respiratory diseases (CRD, mycoplasmosis) [Frequently]

_______ Chronic respiratory diseases (CRD, mycoplasmosis) (14)

How often do you see the diseases/conditions mentioned below on your farm? Please provide an answ... = Coccidiosis [Frequently]

_______ Coccidiosis (15)

How often do you see the diseases/conditions mentioned below on your farm? Please provide an answ... = Worms (Ascaridia, tapeworms) [Frequently]

_______ Worms (Ascaridia, tapeworms) (17)

How often do you see the diseases/conditions mentioned below on your farm? Please provide an answ... = Lice infestation [Frequently]

_______ Lice infestation (18)

How often do you see the diseases/conditions mentioned below on your farm? Please provide an answ... = Mycotoxins (Aflatoxicosis) [Frequently]

_______ Mycotoxicosis (Aflatoxicosis) (19)

How often do you see the diseases/conditions mentioned below on your farm? Please provide an answ... = Nutritional deficiencies [Frequently]

_______ Nutritional deficiencies (20)

How often do you see the diseases/conditions mentioned below on your farm? Please provide an answ... = Egg bound syndrome [Frequently]

_______ Egg bound syndrome (21)

How often do you see the diseases/conditions mentioned below on your farm? Please provide an answ... = Other disease/condition [Frequently]

_______ Other disease/condition on your farm (22)

Display This Question:

If How often do you see the diseases/conditions mentioned below on your farm? Please provide an answ... != Avian infectious bronchitis [Never]

Q94 If **avian infectious bronchitis** occurred on your farm in the past, what clinical signs did you or your vet observe? Please check all boxes that apply

- ☐ Cough (1)
- ☐ Sneeze (2)
- ☐ Tracheal rales (3)
- ☐ Watery eyes (4)
- ☐ Facial swelling (5)
- ☐ Depression (6)
- ☐ Drop in feed consumption (7)
- ☐ Ruffled feathers (8)
- ☐ Egg production drop (9)
- ☐ Wrinkled egg shells (10)
- ☐ Death (11)
- ☐ Not known or do not remember (13)
- ☐ Other, please specify (12) ____

Display This Question:
If How often do you see the diseases/conditions mentioned below on your farm? Please provide an answ... != Avian infectious bronchitis [Never]

*

Q95 How many bird deaths ON AVERAGE were associated with **avian infectious bronchitis** during that production cycle (when infection occurred) on your farm?

____

Display This Question:
If How often do you see the diseases/conditions mentioned below on your farm? Please provide an answ... != Newcastle disease [Never]

Q96 If **Newcastle disease** occurred on your farm in the past, what clinical signs did you or your vet observe? Please check all boxes that apply

- ▢ Cough (1)
- ▢ Sneeze (2)
- ▢ Tracheal rales (3)
- ▢ Watery eyes (4)
- ▢ Facial swelling (5)
- ▢ Tremors (6)
- ▢ Paralysed wings and legs (7)
- ▢ Twisted neck (8)
- ▢ Circling (9)
- ▢ Spasms (10)
- ▢ Depression (11)
- ▢ Watery diarrhoea (12)
- ▢ Drop in feed consumption (13)
- ▢ Egg production drop (14)
- ▢ Death (15)
- ▢ Other, please specify (16) ____

Display This Question:
If How often do you see the diseases/conditions mentioned below on your farm? Please provide an answ... != Newcastle disease [Never]

Q97 How many bird deaths <u>ON AVERAGE</u> were associated with **Newcastle disease** during that production cycle (when infection occurred) on your farm?

____

Display This Question:
If How often do you see the diseases/conditions mentioned below on your farm? Please provide an answ... != Gumboro (infectious bursal disease) [Never]

Q98 If **Gumboro** occurred on your farm in the past, what clinical signs did you or your vet observe? Please check all boxes that apply

- ▢ Severe prostration (1)
- ▢ Incoordination (2)
- ▢ Watery diarrhoea (3)
- ▢ Soiled vent feathers (4)
- ▢ inflammation of the cloaca (5)
- ▢ Drop in weight (6)
- ▢ Death (7)
- ▢ Other, please specify (8) ____

Display This Question:

If How often do you see the diseases/conditions mentioned below on your farm? Please provide an answ... != Gumboro (infectious bursal disease) [Never]

Q99 How many bird deaths <u>ON AVERAGE</u> were associated with **Gumboro** during that production cycle (when infection occurred) on your farm?

____

End of Block: Flock health information

Start of Block: Pharmaceutical products information

Q100 Did you use antibiotics products in the <u>previous</u> production cycle to treat birds on your farm?

- ◯ No (1)
- ◯ Yes (2)
- ◯ Not known or do not remember (3)
- ◯ Not applicable (4)

Display This Question:

If Did you use antibiotics products in the previous production cycle to treat birds on your farm? = Yes

Q101 What **antibiotics** products did you use in the <u>previous</u> production cycle? Please check all boxes that apply

- ☐ Anicyline - Penicillin & Streptomycin (Animal Care) (1)
- ☐ Biaflox - Enrofloxacin (Biacom) (2)
- ☐ Centregent - Gentamicin (Centre) (3)
- ☐ Ciprovet - Ciprofloxacine (Globavet) (4)
- ☐ Conflox -Enrofloxacin (Animal care) (5)
- ☐ Doxygen - Doxycycline & Gentamicin (Kepro) (6)
- ☐ Enrocoli-Max (enrofloxacin + colistin) (7)
- ☐ Enrovet - Enrofloxacin (Globavet) (8)
- ☐ Floxinol - Enrofloxacin (Jubili Agrotech) (9)
- ☐ Flumetin. E.t.c - Augmentin (10)
- ☐ Fura - Furathadol (11)
- ☐ Gentylo - Gentamycin &Tylosin (Centre) (12)
- ☐ Kenflox- Enrofloxacine (Kepro) (13)
- ☐ Kepro-Oxy 5% - Oxytetracycline (14)
- ☐ Neoceryl - Neomycin & Multivitamins (Animal Care) (15)
- ☐ Norflox - Norfloxacine (16)
- ☐ Oxytetracycline 50% - Oxytetracycline (17)
- ☐ SulfaThrim - Sulfaquinoxaline & Trimethoprim (18)
- ☐ Tetracin - Oxytetracycline (Jubili Agrotech) (19)
- ☐ Tylosin - Tylosin (20)
- ☐ Vapcogent - Gentamin (Vapco) (21)
- ☐ Other, please specify (22) ____

Display This Question:

If Did you use antibiotics products in the previous production cycle to treat birds on your farm? = Yes

X→

Q102 For how long did you use the selected antibiotics in your flock **during the previous production cycle if known**? Please select the duration.

	<3 days treatment (1)	3-5 days treatment (2)	6-7 days treatment (3)	>7 days treatment (4)	Do not know (5)
What antibiotics products did you use in the previous production cycle? Please check all boxes th... = Anicyline - Penicillin & Streptomycin (Animal Care) Anicyline - Penicilline & Streptomycin (Animal Care) (17)	○	○	○	○	○
What antibiotics products did you use in the previous production cycle? Please check all boxes th... = Biaflox - Enrofloxacin (Biacom) Biaflox - Enrofloxacin (Biacom) (9)	○	○	○	○	○
What antibiotics products did you use in the previous production cycle? Please check all boxes th... = Centregent - Gentamicin (Centre) Centregent - Gentamicin (Centre) (13)	○	○	○	○	○
What antibiotics products did you use in the previous production cycle? Please check all boxes th... = Ciprovet - Ciprofloxacine (Globavet) Ciprovet - Ciprofloxacine (Globavet) (21)	○	○	○	○	○
What antibiotics products did you use in the previous production cycle? Please check all boxes th... = Conflox -Enrofloxacin (Animal care) Conflox -Enrofloxacin (Animal care) (5)	○	○	○	○	○
What antibiotics products did you use in the previous production cycle? Please check all boxes th... = Enrocoli-Max (enrofloxacin + colistin) Enrocoli-Max (enrofloxacin + colistin) (26)	○	○	○	○	○
What antibiotics products did you use in the previous production cycle? Please check all boxes th... = Enrovet - Enrofloxacin (Globavet) Enrovet - Enrofloxacin (Globavet) (7)	○	○	○	○	○

What antibiotics products did you use in the previous production cycle? Please check all boxes th... = Doxygen - Doxycycline & Gentamicin (Kepro) Doxygen - Doxycycline & Gentamicin (Kepro) (18)	○	○	○	○	○
What antibiotics products did you use in the previous production cycle? Please check all boxes th... = Floxinol - Enrofloxacin (Jubili Agrotech) Floxinol - Enrofloxacin (Jubili Agrotech) (6)	○	○	○	○	○
What antibiotics products did you use in the previous production cycle? Please check all boxes th... = Fura - Furathadol Flumetin. E.t.c - Augmentin (24)	○	○	○	○	○
What antibiotics products did you use in the previous production cycle? Please check all boxes th... = Fura - Furathadol Fura - Furathadol (23)	○	○	○	○	○
What antibiotics products did you use in the previous production cycle? Please check all boxes th... = Gentylo - Gentamycin &Tylosin (Centre) Gentylo - Gentamycin &Tylosin (Centre) (14)	○	○	○	○	○
What antibiotics products did you use in the previous production cycle? Please check all boxes th... = Kenflox- Enrofloxacine (Kepro) Kenflox- Enrofloxacine (Kepro) (4)	○	○	○	○	○
What antibiotics products did you use in the previous production cycle? Please check all boxes th... = Kepro-Oxy 5% - Oxytetracycline Kepro-Oxy 5% - Oxytetracycline (19)	○	○	○	○	○
What antibiotics products did you use in the previous production cycle? Please check all boxes th... = Neoceryl - Neomycin & Multivitamins (Animal Care) Neoceryl - Neomycin & Multivitamins (Animal Care) (22)	○	○	○	○	○

What antibiotics products did you use in the previous production cycle? Please check all boxes th... = Norflox - Norfloxacine Norflox - Norfloxacine (20)					
What antibiotics products did you use in the previous production cycle? Please check all boxes th... = Oxytetracycline 50% - Oxytetracycline Oxytetracycline 50% - Oxytetracycline (11)					
What antibiotics products did you use in the previous production cycle? Please check all boxes th... = SulfaThrim - Sulfaquinoxaline & Trimethoprim SulfaThrim - Sulfaquinoxaline & Trimethoprim (8)					
What antibiotics products did you use in the previous production cycle? Please check all boxes th... = Tetracin - Oxytetracycline (Jubili Agrotech) Tetracin - Oxytetracycline (Jubili Agrotech) (10)					
What antibiotics products did you use in the previous production cycle? Please check all boxes th... = Tylosin - Tylosin Tylosin - Tylosin (16)					
What antibiotics products did you use in the previous production cycle? Please check all boxes th... = Vapcogent - Gentamin (Vapco) Vapcogent - Gentamin (Vapco) (12)					
If What antibiotics products did you use in the previous production cycle? Check all that apply Other, please specify Is Not Empty Other antibiotic specified (25)					

Q103 In general, where do you purchase or what is the source of the pharmaceutical products used on your farm? Please check all boxes that apply.

- ☐ Vet shops (1)
- ☐ My veterinarian (2)
- ☐ Vet pharmaceutical distributors (3)
- ☐ Other, please specify (4) ____

Q104 Where are the sources of the pharmaceutical products used on your farm based?

	Rural area (1)	Village (2)	Town/City (3)
In general, where do you purchase or what is the source of the pharmaceutical products used on yo... = Vet shops Vet shops (19)	☐	☐	☐
In general, where do you purchase or what is the source of the pharmaceutical products used on yo... = My veterinarian My veterinarian (20)	☐	☐	☐
In general, where do you purchase or what is the source of the pharmaceutical products used on yo... = Vet pharmaceutical distributors Vet pharmaceutical distributors (22)	☐	☐	☐
In general, where do you purchase or what is the source of the pharmaceutical products used on yo... = Other, please specify Other type of source (21)	☐	☐	☐

Q105 What is the average amount of time it takes from your farm location to the source (vet-shop, etc.) where you routinely purchase or access pharmaceutical products?

- ◯ less than 60 minutes (2)
- ◯ between 1 and 2 hours (3)
- ◯ between 2 and 3 hours (4)
- ◯ > 3 hours (5)
- ◯ Not known (6)
- ◯ Other, please specify (7) ____

Use Reusable Choices

Q106 Do you experience challenges when obtaining pharmaceutical products needed for farm use?

- ◯ No (x1)
- ◯ Yes (x2)
- ◯ Not known (x3)
- ◯ Not applicable (x4)

Display This Question:
If If Do you experience challenges when obtaining pharmaceutical products needed for farm use? Yes Is Selected

Q107 If response is yes, describe the challenges experienced:

____
____
____
____
____

Use Reusable Choices

Q108 Do you experience challenges related to use of pharmaceutical products on your farm?

- ◯ No (x1)
- ◯ Yes (x2)
- ◯ Not known (x3)
- ◯ Not applicable (x4)

Display This Question:
If If Do you experience challenges related to use of pharmaceutical products on your farm? Yes Is Selected

Q109 If response is yes, name the products (if possible), and describe the challenges experienced:

____
____
____
____
____

Q110 Have you used any **Zoetis (*formerly Pfizer Animal Health*)** pharmaceutical products on your farm in the past?

- ◯ No (1)
- ◯ Yes (2)
- ◯ Not known (3)

Display This Question:
If Have you used any Zoetis (formerly Pfizer Animal Health) pharmaceutical products on your farm in... = Yes

Q111 Which **Zoetis** products have you used? Please provide the name of the products.

	Name of product (5)
Product 1 (1)	
Product 2 (2)	
Product 3 (3)	
Product 4 (4)	
Product 5 (5)	

End of Block: Pharmaceutical products information

Start of Block: Financial costs/profit margin information

Q112 What was the average number of chicks at **beginning of the previous production cycle** on your farm (if applicable)? (PS = parent stock)

	Number of chicks (1)
PS chicks (4)	
Broiler chicks (5)	
Pullet chicks (6)	
Cockerel chicks (7)	

Display This Question:
If Do you have a hatchery on your farm or in another location? = Yes

Q113 What was the **average number of chicks hatched** at your hatchery/hatcheries on your farm **in the previous production cycle** (if applicable)? (PS = parent stock)

	Number of chicks (1)
If farm has a hatchery, what is the type of chicks hatched at the hatchery in the previous produc... = PS chicks PS chicks (4)	
If farm has a hatchery, what is the type of chicks hatched at the hatchery in the previous produc... = Broiler chicks Broiler chicks (5)	
If farm has a hatchery, what is the type of chicks hatched at the hatchery in the previous produc... = Pullet chicks Pullet chicks (6)	
If farm has a hatchery, what is the type of chicks hatched at the hatchery in the previous produc... = Cockerel chicks Cockerel chicks (7)	

Display This Question:

If Do you have a hatchery on your farm or in another location? = Yes

*

Q114 On average, how many **hatchings** occurred on your hatchery/hatcheries **in the previous year** (if applicable)?

____

Display This Question:

If Do you have a hatchery on your farm or in another location? = No

Q115 What was the average cost (Naira) of a one-day old chick hatched or purchased for raising on your farm in the previous production/hatching cycle (if applicable)? (PS = parent stock)

	Average cost (Naira) (1)
PS chicks (4)	
Broiler chicks (5)	
Pullet chicks (6)	
Cockerel chicks (7)	

Display This Question:

If What types of chickens are kept on your farm at present (as of visit date)? (PS = parent stock, G... = Broilers (chicks or adult birds)

*

Q116 What was the estimated **average number of broiler birds that reached slaughter weight** in the previous production cycle (if applicable)?

____

Display This Question:

If What types of chickens are kept on your farm at present (as of visit date)? (PS = parent stock, G... = Broilers (chicks or adult birds)

Q117 What was the **average slaughter age and slaughter weight** of the broilers on your farm through the previous production cycle?

	Please populate the empty boxes (2)
Slaughter age (weeks) (1)	
Slaughter weight (kg) (4)	

Display This Question:
If What types of chickens are kept on your farm at present (as of visit date)? (PS = parent stock, G... = Broilers (chicks or adult birds)

*

Q118 On average, **how many broiler bird mortalities** occured on your farm during the previous production cycle (if applicable)?

____

Display This Question:
If What types of chickens are kept on your farm at present (as of visit date)? (PS = parent stock, G... = Broilers (chicks or adult birds)

*

Q119 What was the **estimated average price** for a broiler bird **at ready slaughter weight** in the previous production cycle (if applicable)?

____

Display This Question:
If What types of chickens are kept on your farm at present (as of visit date)? (PS = parent stock, G... = Layers (chicks or adult birds)

*

Q120 On average what was **the number of layer birds reaching maturity** during the previous production cycle (if applicable)?

____

Display This Question:
If What types of chickens are kept on your farm at present (as of visit date)? (PS = parent stock, G... = Layers (chicks or adult birds)

*

Q121 On average, **how long was the previous production** cycle of **your layer birds** in weeks (if applicable)?

____

Display This Question:
If What types of chickens are kept on your farm at present (as of visit date)? (PS = parent stock, G... = Layers (chicks or adult birds)

*

Q122 What was t**he average number of eggs produced per hen per day** in the previous production cycle on your farm (if applicable)?

____

Display This Question:
If What types of chickens are kept on your farm at present (as of visit date)? (PS = parent stock, G... = Layers (chicks or adult birds)

Q123 What was the **estimated average price PER CRATE for layer eggs** sold during the previous production cycle (if applicable)?

____

Display This Question:
If What types of chickens are kept on your farm at present (as of visit date)? (PS = parent stock, G... = Layers (chicks or adult birds)

Q124 On average, how **many layer bird mortalities** occured on your farm during the previous production cycle (if applicable)?

____

Display This Question:
If What types of chickens are kept on your farm at present (as of visit date)? (PS = parent stock, G... = Layers (chicks or adult birds)

Q125 On average what was **the number of spent-layer or off-layer birds sold for slaughter** at end of the previous production cycle (where applicable)?

____

Q126 On average, how many **unproductive** birds did you remove from your farm during the previous production cycle?
(PS = parent stock, GPS = grand-parent stock)

	Please specify the number (1)
What types of chickens are kept on your farm at present (as of visit date)? (PS = parent stock, G... = Layers (chicks or adult birds)<br>Layers (chicks or adult birds) (293)	
What types of chickens are kept on your farm at present (as of visit date)? (PS = parent stock, G... = Broilers (chicks or adult birds)<br>Broilers (chicks or adult birds) (294)	
What types of chickens are kept on your farm at present (as of visit date)? (PS = parent stock, G... = Breeders-broiler (GPS chicks or adult birds)<br>Breeders-broiler (GPS chicks or adult birds) (295)	
What types of chickens are kept on your farm at present (as of visit date)? (PS = parent stock, G... = Breeders-broiler (PS chicks or adult birds)<br>Breeders-broiler (PS chicks or adult birds) (298)	
What types of chickens are kept on your farm at present (as of visit date)? (PS = parent stock, G... = Breeders-layers (GPS chicks or adult birds)<br>Breeders-layers (GPS chicks or adult birds) (296)	
What types of chickens are kept on your farm at present (as of visit date)? (PS = parent stock, G... = Breeders-layers (PS chicks or adult birds)<br>Breeders-layers (PS chicks or adult birds) (299)	
What types of chickens are kept on your farm at present (as of visit date)? (PS = parent stock, G... = Free-ranging chickens<br>Free-ranging chickens (300)	

What types of chickens are kept on your farm at present (as of visit date)? (PS = parent stock, G... = Cockerels<br>Cockerels (301)	
What types of chickens are kept on your farm at present (as of visit date)? (PS = parent stock, G... = Other type, please specify<br>Other type of bird (297)	

Display This Question:
If What types of chickens are kept on your farm at present (as of visit date)? (PS = parent stock, G... = Layers (chicks or adult birds)

Q127 What was the **estimated average price for spent-layer or off-layer birds** sold for slaughter at end of the previous production cycle?

____

Display This Question:
If Indicate if these activities take place on your farm/premises. Please check all boxes that apply = Operate a slaughter house
Or Indicate if these activities take place on your farm/premises. Please check all boxes that apply = Operate a slaughter house and processing plant

Q128 If farm operates a slaughter house and/or processing plant, how many birds **ON AVERAGE** are slaughtered per week?

____

Display This Question:
If Indicate if these activities take place on your farm/premises. Please check all boxes that apply = Operate a slaughter house
Or Indicate if these activities take place on your farm/premises. Please check all boxes that apply = Operate a slaughter house and processing plant

Q129 If farm operates a slaughter house and/or processing plant, **how many kilograms** ON AVERAGE of the following **NON-packaged** products are produced per week?

	Kg produced (1)
Whole fresh carcass (kg) (1)	
Meat cuts (kg) (2)	
Gizzard (kg) (3)	
Intestine (kg) (4)	
Heads and feet (kg) (5)	

Display This Question:

If Indicate if these activities take place on your farm/premises. Please check all boxes that apply = Operate a slaughter house

Or Indicate if these activities take place on your farm/premises. Please check all boxes that apply = Operate a slaughter house and processing plant

Q130 If farm operates a slaughter house and/or processing plant, what is the AVERAGE cost per kg for the following **NON-packaged** products?

	Cost per kg (1)
Whole fresh carcass (1)	
Meat cuts (2)	
Gizzard (3)	
Intestine (4)	
Heads and feet (5)	

Display This Question:

If Indicate if these activities take place on your farm/premises. Please check all boxes that apply = Operate a slaughter house

Or Indicate if these activities take place on your farm/premises. Please check all boxes that apply = Operate a slaughter house and processing plant

Q131 If farm operates a slaughter house and/or processing plant, **how many kilograms** ON AVERAGE of the following **packaged** products are produced per week?

	Kg produced (1)
Whole fresh carcass (kg) (1)	
Meat cuts (kg) (2)	
Gizzard (kg) (3)	
Heads and feet (kg) (5)	

Display This Question:

If Indicate if these activities take place on your farm/premises. Please check all boxes that apply = Operate a slaughter house

Or Indicate if these activities take place on your farm/premises. Please check all boxes that apply = Operate a slaughter house and processing plant

Q132 If farm operates a slaughter house and/or processing plant, what is the AVERAGE cost per kg for the following **packaged** products?

	Cost per kg (1)
Whole fresh carcass (1)	
Meat cuts (2)	
Gizzard (3)	
Heads and feet (5)	

Display This Question:
If Indicate if these activities take place on your farm/premises. Please check all boxes that apply = Operate a slaughter house
Or Indicate if these activities take place on your farm/premises. Please check all boxes that apply = Operate a slaughter house and processing plant

*

Q133 If farm operates a slaughter house and/or processing plant, what is the estimated cost of running the unit per month (electricity, water, etc) (if applicable)?

____

Q134 What was the estimated cost of **feeding** on your farm through the previous production cycle (where applicable)?

	What types of chickens are kept on your farm at present (as of visit date)? (PS = parent stock, G... = Broilers (chicks or adult birds) Broilers (4)	*What types of chickens are kept on your farm at present (as of visit date)? (PS = parent stock, G... = Layers (chicks or adult birds)* Layers (5)
Cost of feeding (7)		

Q135 Who performs the vaccinations on your farm? Please select all that apply

- ▢ Farm owner or farm worker (1)
- ▢ Contracted vet (2)
- ▢ Other contracted animal health workers (3)
- ▢ Other, please specify (4) ____

Q136 If known, what was the estimated cost of **<u>all</u> antibiotic treatments** provided on your farm through <u>the previous production cycle?</u>

____

Q137 If known, what was the **estimated average cost of the vaccination program** including cost of vaccines, vet (if applicable) and material for vaccination on your farm through the <u>previous</u> production cycle?

____

Q138 If known, what was the estimated average cost of putting in place and maintaining **biosecurity practices/procedures** (including washing hands with soap, using foot baths filled with disinfectant, safe disposal of dead birds) performed on your farm through <u>the previous production cycle</u>?

____

Q139 If known, what was the estimated average cost of **litter management** on your farm through <u>the previous production cycle</u>?

____

Q140 If known, what is the estimated average cost of **staff wages and benefits** (manager, farm workers, etc) on your farm <u>annually</u>?

____

Q141 If known, what is the estimated average cost of **waste disposal staff wages and benefits** (dead birds, litter, other, etc) on your farm <u>annually</u>?

____

End of Block: Financial costs/profit margin information

Start of Block: Other information

Q142 In your opinion, what do you consider as the top two key challenges faced on your farm in relation to a vaccination program?

____
____
____
____

Q143 In your opinion, what do you consider as the top two key challenges faced on your farm in relation to availability of pharmaceutical products needed for farm use?

Q144 In your opinion, what do you consider as the top two key challenges faced on your farm in relation to flock/bird health or diseases?

Q145 In your opinion, what do you consider as the top two key challenges faced on your farm in relation to feeding and watering?

Q146 In your opinion, what do you consider as the top two key challenges faced on your farm in relation to housing and litter management?

End of Block: Other information
