# Supplementary material for: Baseline Assessment of Poultry Production, Pharmaceutical Product Use, and Related Challenges on Commercial Poultry Flocks in Kano and Oyo States of Nigeria
Source: Vet Sci. 2021 Dec 8;8(12):315. doi: 10.3390/vetsci8120315 (PMC8709114; doi:10.3390/vetsci8120315)
Supplement: Supplementary file 1 [file vetsci-08-00315-s001.zip › supplementary/Tables S1-S9.pdf]

Supplementary Material

Table S1. Respondent demographics and general farm characteristics as presented in Ekiri *et al.*, unpublished.

**Variable**	**Response**	**Total respondents (n%) (*n*=43)**	**Respondents in Kano (n%) (*n*=27)**	**Respondents in Oyo (n%) (*n*=16)**
Age[1]	Under 35 years	6 (14.3)	3 (11.5)	3 (18.8)
	35 – 45 years	12 (28.6)	10 (38.5)	2 (12.5)
	46 – 60 years	20 (47.6)	11 (42.3)	9 (56.3)
	Over 60 years	4 (9.5)	2 (7.7)	2 (12.5)
Gender[1]	Male	30 (71.4)	16 (61.5)	14 (87.5)
	Female	12 (28.6)	10 (38.5)	2 (12.5)
Years of experience in poultry farming	0-5 years	7 (16.3)	4 (14.8)	3 (18.8)
	6-10 years	12 (27.9)	7 (25.9)	5 (31.3)
	11-20 years	16 (37.2)	12 (44.4)	4 (25.0)
	Over 20 years	8 (18.6)	4 (14.8)	4 (25.0)
Current role on farm[2]	Farm owner	29 (67.4)	21 (77.8)	8 (50.0)
	Farm manager	16 (37.2)	8 (29.6)	8 (50.0)
	Farm supervisor	0	0	0
	Farm worker	0	0	0
	Farm vet	0	0	0
	Other	0	0	0
Years farm has been in existence	0-5 years	13 (30.2)	8 (29.6)	5 (31.3)
	6-10 years	19 (44.2)	11 (40.7)	8 (50.0)
	11-20 years	9 (20.9)	7 (25.9)	2 (12.5)
	Over 20 years	2 (4.7)	1 (3.7)	1 (6.3)
Types of chickens kept on farm at present [2]	Layers only [3] (chicks or adult birds)	21 (48.8)	13 (48.1)	8 (50.0)
	Broilers only [4] (chicks or adult birds)	9 (20.9)	7 (25.9)	2 (12.5)
	Layers and broilers [5] (chicks or adult birds)	12 (27.9)	7 (25.9)	5 (31.3)
	Breeders [6]	2 (4.7)	0	2 (12.5)
Membership in poultry association at different levels[2]	Poultry association at zonal/local government	8 (18.6)	-	-
	Poultry association at state level	30 (69.8)	-	-
	Poultry association at national level	3 (7.0)	-	-
	Other	5 (11.6)	-	-

[1] There were missing values for these variables: age group (n=1) and gender (n=1). [2] Respondents selected all applicable responses to these variables; column percentages may not add up to 100%. [3] Within the layer farms (n=21): n=4 also kept cockerels and n=1 kept other bird types (local chickens). [4] Within the broiler farms (n=9): n=1 also kept other bird types (local chickens and guinea fowl). [5] Within the mixed (layers and broilers) farms (n=12): n=1 kept breeder-broilers and breeder-layers (PS chicks/adults), n=1 also kept free-ranging chickens, n=4 kept cockerels, and n=1 kept other bird types (local chickens and guinea fowl). [6] Farms that kept breeders (n=2) included: one mixed farm (i.e., had both layers and breeders) that kept both breeder-broilers (PS chicks/adults) and breeder-layers (PS chicks/adults), and one farm that kept breeder-broilers only. Abbreviations: PS: Parent-stock.

Table S2. Average number of chicks at the beginning of the previous production cycle on farm (if applicable).

Average number of chicks	State	n	Mean	SD	Median	Min	Max
PS chicks	Total	2	5,500	2,121	5,500	4,000	7,000
	Oyo	2	5,500	2,121	5,500	4,000	7,000
	Kano	0	NA	NA	NA	NA	NA
Broiler chicks	Total	15	1,344	1,402	500	100	5,000
	Oyo	4	2,565	2,007	2,530	200	5,000
	Kano	11	900	855	500	100	2,500
Pullet chicks	Total	35	5,531	5,380	4,000	300	26,000
	Oyo	13	6,580	4,232	4,377	1,500	12,960
	Kano	22	4,911	5,962	3,250	300	26,000
Cockerel chicks	Total	5	174	195	100	20	500
	Oyo	1	20	NA	20	20	20
	Kano	4	213	202	150	50	500

Abbreviations: NA, not applicable; PS, parent stock; SD, standard deviation.

Table S3. Average cost (NGN) of a one-day old chick hatched or purchased for raising on farm in the previous production/hatching cycle (if applicable).

Average cost (NGN) of a one-day old chick	State	n	Mean	SD	Median	Min	Max
PS chicks	Total	0	NA	NA	NA	NA	NA
	Oyo	0	NA	NA	NA	NA	NA
	Kano	0	NA	NA	NA	NA	NA
Broiler chicks	Total	15	302	183	270	120	920
	Oyo	3	463	398	280	190	920
	Kano	12	262	69	270	120	340
Pullet chicks	Total	31	2,406	11,623	200	120	65,000
	Oyo	9	600	625	220	170	1,700
	Kano	22	3,145	13,815	200	120	65,000
Cockerel chicks	Total	5	223	434	30	25	1,000
	Oyo	1	1,000	NA	1,000	1,000	1,000
	Kano	4	29	3	30	25	30

Abbreviations: NA, not applicable; NGN, Nigerian Naira; PS, parent stock; SD, standard deviation. At the time of writing 1 US dollar = 411.400 NGN (August 2021).

Table S4. Average number of chicks hatched at hatchery/hatcheries on-farm in the previous production cycle (if applicable).

Average number of chicks hatched in the previous production cycle	n	Mean	SD	Median	Min	Max
PS chicks	0	NA	NA	NA	NA	NA
Broiler chicks	2	10,000	0	10,000	10,000	10,000
Pullet chicks	1	15,000	NA	15,000	15,000	15,000
Cockerel chicks	1	10,000	NA	10,000	10,000	10,000

Abbreviations: NA, not applicable; PS, parent stock; SD, standard deviation.

Table S5. Number of unproductive birds culled from farm during the previous production cycle.

Average number of unproductive birds culled	State	n	Mean	SD	Median	Min	Max
Layer	Total	27	381	1,082	30	0	5,228
	Oyo	10	24	27	15	0	75
	Kano	17	590	1,332	34	0	5,228
Broiler	Total	14	24	19	18	0	70
	Oyo	4	15	4	14	10	20
	Kano	10	27	22	20	0	70
GPS broiler	Total	0	NA	NA	NA	NA	NA
	Oyo	0	NA	NA	NA	NA	NA
	Kano	0	NA	NA	NA	NA	NA
PS broiler	Total	1	15	NA	15	15	15
	Oyo	1	15	NA	15	15	15
	Kano	0	NA	NA	NA	NA	NA
GPS layer	Total	0	NA	NA	NA	NA	NA
	Oyo	0	NA	NA	NA	NA	NA
	Kano	0	NA	NA	NA	NA	NA
PS layer	Total	0	NA	NA	NA	NA	NA
	Oyo	0	NA	NA	NA	NA	NA
	Kano	0	NA	NA	NA	NA	NA
Free-range chickens	Total	0	NA	NA	NA	NA	NA
	Oyo	0	NA	NA	NA	NA	NA
	Kano	0	NA	NA	NA	NA	NA
Cockerel	Total	4	6	9	3	0	20
	Oyo	0	NA	NA	NA	NA	NA
	Kano	4	6	9	3	0	20
Other birds	Total	0	NA	NA	NA	NA	NA
	Oyo	0	NA	NA	NA	NA	NA
	Kano	0	NA	NA	NA	NA	NA

Abbreviations: GPS, grandparent stock; NA, not applicable; PS, parent stock; SD, standard deviation.

Table S6. Estimated cost of vaccines (NGN) per 1000 birds in the previous production cycle.

**Disease vaccinated against**			**n**	**Mean**	**SD**	**Median**	**Min**	**Max**
Infectious bronchitis		Total	3	1,683	325	1,700	1,350	2,000
	**State**	Oyo	3	1,683	325	1,700	1,350	2,000
		Kano	0	NA	NA	NA	NA	NA
	**Farm type**	Layers	0	NA	NA	NA	NA	NA
		Broilers	0	NA	NA	NA	NA	NA
		Mixed [1]	3	1,683	325	1,700	1,350	2,000
Marek's disease		Total	13	5,423	1,739	5,000	2,200	9,000
	**State**	Oyo	3	3,900	1,493	4,500	2,200	5,000
		Kano	10	5,880	1,592	6,000	4,000	9,000
	**Farm type**	Layers	6	5,367	2,260	5,250	2,200	9,000
		Broilers	1	4,800	NA	4,800	4,800	4,800
		Mixed	6	5,583	1,429	5,500	4,000	8,000
Newcastle disease		Total	35	7,524	8,388	2,500	950	26,200
	**State**	Oyo	8	1,304	465	1,000	950	2,000
		Kano	27	9,367	8,747	4,900	1,100	26,200
	**Farm type**	Layers	16	11,597	9,459	9,600	950	26,200
		Broilers	8	3,638	5,116	1,750	1,200	16,200
		Mixed	10	4,718	6,371	1,850	980	21,500
Newcastle disease and		Total	5	4,500	4,868	2,500	2,000	13,200
Infectious bronchitis	**State**	Oyo	5	4,500	4,868	2,500	2,000	13,200
		Kano	0	NA	NA	NA	NA	NA
	**Farm type[2]**	Layers	3	6,000	6,236	2,500	2,300	13,200
		Broilers	0	NA	NA	NA	NA	NA
		Mixed	1	2,500	NA	2,500	2,500	2,500
Newcastle disease and		Total	17	25,353	12,649	22,000	15,000	70,000
Infectious bronchitis and	**State**	Oyo	8	26,625	18,654	19,000	15,000	70,000
Egg drop syndrome		Kano	9	24,222	3,528	25,000	19,000	30,000
	**Farm type**	Layers	9	26,889	16,930	24,000	15,000	70,000
		Broilers	0	NA	NA	NA	NA	NA
		Mixed	7	24,143	5,872	22,000	18,000	35,000
Newcastle disease and		Total	1	21,000	NA	21,000	21,000	21,000
Egg drop syndrome	**State**	Oyo	1	21,000	NA	21,000	21,000	21,000
		Kano	0	NA	NA	NA	NA	NA
	**Farm type**	Layers	1	21,000	NA	21,000	21,000	21,000
		Broilers	0	NA	NA	NA	NA	NA
		Mixed	0	NA	NA	NA	NA	NA
Newcastle disease and		Total	2	17,500	3,536	17,500	15,000	20,000
Infectious coryza	**State**	Oyo	1	15,000	NA	15,000	15,000	15,000
		Kano	1	20,000	NA	20,000	20,000	20,000
	**Farm type**	Layers	1	20,000	NA	20,000	20,000	20,000
		Broilers	0	NA	NA	NA	NA	NA
		Mixed	0	NA	NA	NA	NA	NA
Infectious bursal disease		Total	32	1,984	1,019	1,750	600	5,000
	**State**	Oyo	7	2,120	1,437	1,500	920	5,000
		Kano	25	1,946	905	1,800	600	5,000
	**Farm type**	Layers	16	1,818	646	1,800	900	3,600
		Broilers	8	2,675	1,659	2,200	600	5,000
		Mixed	7	1,643	479	1,700	1,000	2,500

Table S6. Continued.

Disease vaccinated against			n	Mean	SD	Median	Min	Max
Avian encephalomyelitis		Total	1	5,000	NA	5,000	5,000	5,000
	**State**	Oyo	1	5,000	NA	5,000	5,000	5,000
		Kano	0	NA	NA	NA	NA	NA
	**Farm type**	Layers	0	NA	NA	NA	NA	NA
		Broilers	0	NA	NA	NA	NA	NA
		Mixed	0	NA	NA	NA	NA	NA
Egg drop syndrome		Total	3	12,667	4,041	15,000	8,000	15,000
	**State**	Oyo	3	12,667	4,041	15,000	8,000	15,000
		Kano	0	NA	NA	NA	NA	NA
	**Farm type**	Layers	1	15,000	NA	15,000	15,000	15,000
		Broilers	0	NA	NA	NA	NA	NA
		Mixed	1	8,000	NA	8,000	8,000	8,000
Fowl pox		Total	27	2,236	1,299	2,200	500	8,000
	**State**	Oyo	6	3,047	2,708	2,750	500	8,000
		Kano	21	2,005	330	2,100	1,300	2,600
	**Farm type**	Layers	15	1,993	658	2,100	500	3,500
		Broilers	2	2,000	424	2,000	1,700	2,300
		Mixed	9	2,053	626	2,200	780	3,000
Colibacillosis		Total	3	7,167	3,819	8,000	3,000	10,500
	**State**	Oyo	1	3,000	NA	3,000	3,000	3,000
		Kano	2	9,250	1,768	9,250	8,000	10,500
	**Farm type**	Layers	2	9,250	1,768	9,250	8,000	10,500
		Broilers	0	NA	NA	NA	NA	NA
		Mixed	1	3,000	NA	3,000	3,000	3,000
Fowl cholera (pasteurellosis) and fowl typhoid (salmonellosis)		Total	1	1,400	NA	1,400	1,400	1,400
	**State**	Oyo	1	1,400	NA	1,400	1,400	1,400
		Kano	0	NA	NA	NA	NA	NA
	**Farm type**	Layers	1	1,400	NA	1,400	1,400	1,400
		Broilers	0	NA	NA	NA	NA	NA
		Mixed	0	NA	NA	NA	NA	NA
Mycoplasmosis		Total	3	6,000	7,795	1,600	1,400	15,000
	**State**	Oyo	3	6,000	7,795	1,600	1,400	15,000
		Kano	0	NA	NA	NA	NA	NA
	**Farm type**	Layers	1	1,400	NA	1,400	1,400	1,400
		Broilers	1	15,000	NA	15,000	15,000	15,000
		Mixed	1	1,600	NA	1,600	1,600	1,600
Coccidiosis		Total	3	6,200	4,257	7,000	1,600	10,000
	**State**	Oyo	3	6,200	4,257	7,000	1,600	10,000
		Kano	0	NA	NA	NA	NA	NA
	**Farm type**	Layers	0	NA	NA	NA	NA	NA
		Broilers	1	10,000	NA	10,000	10,000	10,000
		Mixed	1	1,600	NA	1,600	1,600	1,600
Avian encephalomyelitis and Fowl pox [3]		Total	0	NA	NA	NA	NA	NA
Tenosynovitis [3]		Total	0	NA	NA	NA	NA	NA
Other vaccinations [3]		Total	0	NA	NA	NA	NA	NA

[1]Mixed farms kept both layers and broilers. [2] Numbers by farm type may not add up to the total as there were two farms that kept breeders only (these were not included in any of the other farm categories).[3]Analysis by state and farm type are not shown for these diseases, as the total number of respondents was zero. Abbreviations: NA, not applicable; NGN, Nigerian Naira; SD, standard deviation. At the time of writing 1 US dollar = 411.400 NGN (August 2021).

Table S7. Antibiotics used on the farm during the previous production cycle.

Did you use antibiotics products in the previous production cycle to treat birds on your farm?	Total respondents (n%) (n=43)	Respondents in Kano (n%) (n=27)	Respondents in Oyo (n%) (n=16)
No	1 (2.3)	0	1 (6.3)
Yes	42 (97.7)	27 (100)	15 (93.7)

What antibiotics products did you use in the previous production cycle? Local commercial name (manufacturer)	Antimicrobial	Total respondents (n%) (n=42)[1]	Respondents in Kano (n%) (n=27)[1]	Respondents in Oyo (n%) (n=15)[1]
Doxygen (Kepro)	Doxycycline & Gentamicin	27 (64.3)	16 (59.3)	11 (73.3)
Tylosin (not specified)	Tylosin	25 (59.5)	14 (51.9)	11 (73.3)
Conflox (Animal care)	Enrofloxacin	23 (54.8)	14 (51.9)	9 (60.0)
Neoceryl (Animal care)	Neomycin & Multivitamins	20 (47.6)	15 (55.6)	5 (33.3)
Centregent (Centre)	Gentamicin	19 (45.2)	10 (37.0)	9 (60.0)
Kenflox (Kepro)	Enrofloxacine	18 (42.9)	15 (55.6)	3 (20.0)
Other antibiotics	Other antibiotics	15 (35.7)	14 (51.9)	1 (6.7)
Kepro-Oxy 5% (Kepro)	Oxytetracycline	14 (33.3)	8 (29.6)	6 (40.0)
GenTylo (Centre)	Gentamycin & Tylosin	13 (31.0)	7 (25.9)	6 (40.0)
Ciprovet (Globavet)	Ciprofloxacin	12 (28.6)	8 (29.6)	4 (26.7)
Biaflox (Biacom)	Enrofloxacin	11 (26.2)	2 (7.4)	9 (60.0)
Fura	Furathadol	11 (26.2)	9 (33.3)	2 (13.3)
Oxytetracycline 50%	Oxytetracycline	11 (26.2)	9 (33.3)	2 (13.3)
Anicyline (Animal Care)	Penicillin & Streptomycin	9 (21.4)	5 (18.5)	4 (26.7)
Floxinol (Jubili Agrotech)	Enrofloxacin	7 (16.7)	4 (14.8)	3 (20.0)
Tetracin (Jubili Agrotech)	Oxytetracycline	5 (11.9)	4 (14.8)	1 (6.7)
Enrovet (Globavet)	Enrofloxacin	3 (7.1)	1 (3.7)	2 (13.3)
Norflox	Norfloxacine	3 (7.1)	3 (11.1)	0
Enrocoli-Max	Enrofloxacin & Colistin	2 (4.8)	0	2 (13.3)
Flumetin. E.t.c (Augmentin)	Amoxicillin clavulanate	1 (2.4)	0	1 (6.7)
Vapcogent (Vapco)	Gentamin	1 (2.4)	0	1 (6.7)
SulfaThrim	Sulfaquinoxaline & Trimethoprim	0	0	0

[1] Respondents selected all applicable antibiotics; therefore, column percentages do not add up to 100%.

Table S8. Duration of antibiotic use during the previous production cycle.

For how long did you use the selected antibiotics in your flock during the previous production cycle if known? [1]		Total respondents (n)	< 3 days (n %)	3-5 days (n %)	6-7 days (n %)	>7 days (n %)	Not known (n %)
**Commercial name (manufacturer)**	**Antimicrobial**						
Anicyline (Animal Care)	Penicillin & Streptomycin	9	0	9 (100)	0	0	0
Biaflox (Biacom)	Enrofloxacin	11	0	11 (100)	0	0	0
Centregent (Centre)	Gentamicin	19	0	1 (5.3)	16 (84.2)	2 (10.5)	0
Ciprovet (Globavet)	Ciprofloxacin	12	0	11 (91.7)	1 (8.3)	0	0
Conflox (Animal care)	Enrofloxacin	23	0	22 (95.7)	1 (4.4)	0	0
Doxygen (Kepro)	Doxycycline & Gentamicin	27	0	26 (96.3)	1 (3.7)	0	0
Enrocoli-Max	Enrofloxacin & Colistin	2	0	2 (100)	0	0	0
Enrovet (Globavet)	Enrofloxacin	3	0	3 (100)	0	0	0
Floxinol (Jubili Agrotech)	Enrofloxacin	7	0	6 (85.7)	1 (14.3)	0	0
Flumetin. E.t.c (Augmentin)	Amoxicillin clavulanate	0	0	0	0	0	0
Fura	Furathadol	11	0	11 (100)	0	0	0
GenTylo (Centre)	Gentamycin & Tylosin	13	0	13 (100)	0	0	0
Kenflox (Kepro)	Enrofloxacine	18	0	18 (100)	0	0	0
Kepro-Oxy 5% (Kepro)	Oxytetracycline	14	1 (7.1)	13 (92.9)	0	0	0
Neoceryl (Animal care)	Neomycin & Multivitamins	20	0	20 (100)	0	0	0
Norflox	Norfloxacine	3	0	3 (100)	0	0	0
Oxytetracycline 50%	Oxytetracycline	11	0	11 (100)	0	0	0
SulfaThrim	Sulfaquinoxaline & Trimethoprim	0	0	0	0	0	0
Tetracin (Jubili Agrotech)	Oxytetracycline	5	0	5 (100)	0	0	0
Tylosin (not specified)	Tylosin	24	1 (4.2)	22 (91.7)	1 (4.2)	0	0
Vapcogent (Vapco)	Gentamin	1	0	1 (100)	0	0	0
Other antibiotics	Other antibiotics	15	0	13 (86.7)	1 (6.7)	0	1 (6.7)

[1] Respondents selected responses for each antibiotic used, therefore column percentages do not add up to 100%.

Table S9. Advice on vaccination protocols, source of vaccines purchased by respondents, and person performing the vaccination on farm.

Variable	Response	Total respondents (n%) (n=43)	Respondents in Kano (n%) (n=27)	Respondents in Oyo (n%) (n=16)
Who gives advice on the vaccine protocol used on farm? [1]	My veterinarian	16 (37.2)	8 (29.6)	8 (50.0)
	Government official (vet, paravet or agriculture extension officer)	12 (27.9)	12 (44.4)	0
	No one, I just know what to do	9 (20.9)	3 (11.1)	6 (37.5)
	Other person	5 (11.6)	4 (14.8)	1 (6.3)
	Vet shop attendant	3 (7.0)	2 (7.4)	1 (6.3)
	Other[2]- Other farmers	1 (2.3)	-	-
	Other- Poultry association	1 (2.3)	-	-
	Other- Private paravet	1 (2.3)	-	-
Where do you source the vaccines? (i.e. location of vaccine supplier)[1]	Vet shop	32 (74.4)	21 (77.8)	11 (68.8)
	Vet pharmaceutical distributor	8 (18.6)	3 (11.1)	5 (31.3)
	Veterinarian	3 (7.0)	3 (11.1)	0
	Other person	1 (2.3)	1 (3.7)	0
	Government provides (vet, paravet or agriculture extension officer)	0	0	0
	Other- Importers	1 (2.3)	-	-
Who performs the vaccinations on farm?[1]	Farm owner or farm worker	29 (67.4)	21 (77.8)	8 (50.0)
	Contracted vet	17 (39.5)	9 (33.3)	8 (50.0)
	Other contracted animal health workers	4 (9.3)	4 (14.8)	0
	Other professional	0	0	0

[1]Respondents selected all applicable responses for these variables; column percentages for specified variable do not add up to 100%. [2]Respondents provided optional alternative response.
